# “We are protectors, not protestors”: global impacts of extractivism on human–nature bonds

**DOI:** 10.1007/s11625-024-01526-1

**Published:** 2024-08-23

**Authors:** Ksenija Hanaček, Dalena Tran, Arielle Landau, Teresa Sanz, May Aye Thiri, Grettel Navas, Daniela Del Bene, Juan Liu, Mariana Walter, Aida Lopez, Brototi Roy, Eleonora Fanari, Joan Martinez-Alier

**Affiliations:** 1 Institute of Environmental Science and Technology (ICTA), Autonomous University of Barcelona, Edifici ICTA-ICP, Carrer de les Columnes S/N, Campus de la UAB, 08193 Cerdanyola del Vallès, Barcelona, Spain; 2 Faculty of Social Sciences, Global Development Studies, University of Helsinki, Unioninkatu 35, PO Box 18, 00014 Helsinki, Finland; 3 Graduate School of Frontier Sciences, University of Tokyo, 5-1-5 Kashiwanoha, Kashiwa, Chiba Japan; 4 Facultad de Gobierno, University of Chile, Santa Lucía 240, Santiago, Chile; 5 College of Humanities and Development Studies, China Agricultural University, 2925+WC3, Qinghua E Rd, Haidian District, Beijing, 100107 China; 6 Institut Barcelona d’ Estudis Internacionals, IBEI, Ramon Trias Fargas 25-27, 08005 Barcelona, Spain; 7 Autonomous University of Mexico City, Plantel Del Valle, San Lorenzo 290, Colonial del Valle, 03100 Cuidade de México, México; 8 Department of Environmental Sciences and Policy, Central European University, Vienna, Quellenstraße 51, 1100 Vienna, Austria

**Keywords:** Human–nature bonds, Extractivism, Colonialism, Environmental justice, Sustainability

## Abstract

This article analyzes the global impacts of extractivism on human–nature bonds. To do so, we rely on socio-ecological conflict data from the Global Atlas of Environmental Justice. Over 1800 cases involving resistance to the destruction of nature, cultures, cosmologies, worldviews, ancestral origins, and sacred places are analyzed using log-linear regression compared to 1600 cases that do not report such loss. The impact is especially visible when mineral ores, plantation products, and crude oil are extracted. The results indicate that affected groups are Indigenous peoples, farmers, peasants, pastoralists, and religious groups. In conflict outcomes, 79% of cases with refusal of compensation indicate impacts on human–nature bonds. Furthermore, in those cases where assassinations of activists occurred, 68% have observed impacts on human–nature bonds. Protecting human–nature bonds is a critical component for achieving social, economic, and environmental sustainability and justice against extractivism embedded in colonial relations playing against such bonds and environmental protectors.

**Supplementary Information:**

The online version contains supplementary material available at 10.1007/s11625-024-01526-1.

## Introduction

Extractivism is at the root of many environmental distribution conflicts (Martinez-Alier 2002; Scheidel et al. 2020). Tensions arise when governments or corporations acquire lands for extractivist activities such as large scale mining, mass infrastructure, mega dams, tree plantations, and intensive agriculture for trade and profit purposes (Acosta 2011; Cristina et al. 2012; Kröger and Ehrnström-Fuentes 2021; Mora-Motta et al. 2020; Willow 2016). These projects often involve an enormous physical transformation of the environment, thereby jeopardizing local peoples’ access to and use of natural resources (Peluso and Lund 2011). However, as part of this process, long-standing human–nature bonds, such as ways of being and ways of relating to nature, feeling about nature, and knowing about nature, are systemically affected and subject to change (Escobar 2018; Fernández-Llamazares et al. 2021; McGregor et al. 2020; Rodríguez and Inturias 2018; Willow 2016).

When highly damaging extractivist activities are sited on territories, affected people often mobilize and claim environmental justice (Kröger et al. 2021; O’Connor and Martinez-Alier 1998). In much of the environmental justice literature, however, the question of how human–nature bonds are affected and protected still remains underrepresented, despite their importance for sustainability pathways through social justice, worldviews, culture, local economies, and system beliefs based on nature (Hanaček and Rodríguez-Labajos 2018; Isacs et al. 2022; John 2016; Kojola and Pellow 2020; McGregor et al. 2020; Nygren and Rikoon 2008; Rodríguez and Inturias 2018). Anthropocentric universalism imposed through extractivist projects has been widely challenged, showing how relational ontologies and epistemologies counter mainstream understandings of sustainability (see Kaul et al. 2022, special issue in this journal).

In addition, there has been a wide discussion on the “cultural ecosystem services,” or non-material benefits people obtain from ecosystems, such as esthetic, identity, and spirituality, including debates on their economic valuation (Arias-Arévalo et al. 2017; Chan et al. 2016; Martín-López et al. 2012). The Intergovernmental Science-Policy Platform on Biodiversity and Ecosystem Services (IPBES) has also recognized “nature’s contributions to human society” and plural values (Díaz et al. 2018). This perspective has made important contributions to the literature emphasizing human–nature relationships supporting plural valuations of nature. Similarly, impacts on “cultural ecosystem services” about conflicts over agroecosystems have been recently analyzed (Hanaček and Rodríguez-Labajos 2018). The present study, however, expands this analysis to environmental conflicts related to impacts on human–nature bonds by looking at a large diversity of mutually interconnected micro and macro systems, including forests, trees, plants, animals, rivers, mountains, rocks, seas, oceans, and the cosmos (Kahanamoku et al. 2020; Neale 2017; Virtanen 2022).

Furthermore, the literature has widely acknowledged that extractivist tendencies go hand-in-hand with colonial relations, which in turn perpetuate the dispossession of land along with cultures, knowledge, spirituality, Indigenous self-governance, and self-determination (John 2016; Willow 2016). Thus, colonialism is a substantial precedent for studying environmental conflicts posing a threat to the human–nature bonds people have been protecting for centuries (McGregor et al. 2020; Silan and Munkejord 2022). As Tuhiwai Smith (1999, p. 37) explains,

“Colonialism denies the validity of peoples’ claim to existence, to land and territories, to the right of self-determination, to the survival of their languages and forms of cultural knowledge, including natural resources and systems for living within their environments […]. Although communities, their cultures, languages, and socio-economic practices are often seen as spaces of marginalization, still, these are spaces of resistance.”

Environmental injustices have been studied concerning social class, race, and ethnicity (Pulido 2017; Pulido et al. 2016; Sultana 2020), biophysical aspects, such as energy and materials (O’Connor and Martinez-Alier 1998; Russi et al. 2008), political governance (Muradian et al. 2003), economic aspects (Pérez-Rincón et al. 2019; Verbrugge and Geenen 2019), industrial toxicology and public health (Bullard et al. 2007; Collins et al. 2016; Navas et al. 2022), and global climate change (Sultana 2022). This article, in turn, emphasizes the impact on human–nature bonds as a form of environmental injustice (McGregor et al. 2020). Looking at human–nature bonds in environmental justice conflicts sheds light on the importance of relational nature stewardship as a strategy against extractivism, often colonial or undemocratic and oppressive systems in defense of environmental sustainability and social justice (Chuluu 2021; Escobar 2018).

To this end, this article provides a global assessment of mobilizations against extractivism in cases when human–nature bonds are both central impacts of the damaging projects and arguments to reclaim environmental justice (Hanaček and Rodríguez-Labajos 2018). We do so by looking into cases registered in the Global Atlas of Environmental Justice (EJAtlas). We choose to focus on human–nature bonds put at risk through extractivism because of human–nature importance for local communities and their power in mobilization (Tuhiwai Smith 1999; Whyte 2017a).

## Theoretical framework: political ecology of human–nature bonds

Political ecology as a field addresses questions on how human society and the environment affect each other over time, a notable weakness of other social science disciplines (Walker 1998). It primarily grew out of the traditions of political economy and cultural ecology to answer why and how environmental problems are created and changed over time (ibid). Thus, political ecology is the study of the relationships between political, economic, and social factors with environmental issues and changes. For example, in their work titled *Land Degradation and Society*, Blaikie and Brookfield (1986) explain that any change in land patterns represents a shift in social structure and relationship to the land. The authors emphasize that such a shift leads to affected communities’ impoverishment and marginalization at the hands of public and private industries’ encroachment. Furthermore, research on political ecology and human geography indicate that people, space, place, identities, knowledge, and lived experiences are intersected by a range of changes in biophysical land patterns (Nygren and Rikoon 2008; Pulido and De Lara 2018; Sultana 2014).

Therefore, political ecology is at the confluence between ecologically rooted social science and the principles of political economy, as the field frames every ecological issue as a political one. Politics has to do with the distribution of power and resources within a given group, community, and society, within and across generations. The academic community of political ecology offers a wide range of studies integrating ecological social sciences with political economy (Watts and Peet 1996) in topics such as degradation and marginalization, environmental conflict, conservation and control, environmental identities, and social movements (see Robbins 2004). In addition, the field attempts to provide critiques as well as alternatives in the interplay of the environment and political, economic, and social factors. Robbins (2004, p. 12) asserts that the discipline has a “normative understanding that there are very likely better, less coercive, less exploitative, and more sustainable ways of doing things.” Political ecology does not aim to necessarily generate policies, like environmental politics does, but to understand the phenomenon and engage with social mobilization. In recent years, political ecology has been expanding towards more such critical avenues, including emotional political ecology and human exceptionalism (e.g., González-Hidalgo and Zografos 2020; Srinivasan and Kasturirangan 2016).

This paper builds upon political ecology approaches to understand power relations by analyzing disruptions to the human–nature bonds in environmental conflicts (O’Connor and Martinez-Alier 1998). We additionally consider colonialism embedded in extractivism and industrial development that neglects claims for human–nature bonds, non-dominant modes of knowledge, and land management (McGregor et al. 2020). We follow Escobar (2001, p. 36), who describes links between people and their environments in environmental conflicts as a “power dynamic associated with meaning-making practices and values that regulate social life, local economy, ecology and nature, personhood, knowledge, history, feelings, and emotions.”

Human–nature bonds are place and context specific and go beyond any single definition or research field (Yellow Cloud and Redvers 2023). In this article, we focus on different understandings of human-nature bonds from political ecology and environmentalism theories (Martinez-Alier 2002; Scheidel et al. 2023). This bottom-up social mobilization focus on human–nature bonds is necessary for capturing social injustices in sustainability topics. More broadly, however, human–nature bonds are ways of connectedness to nature as well as forming a web of life on Earth and co-existing with other-than-human nature. Human–nature bonds go beyond the planet Earth and may include connectedness with the Cosmos as well. Thus, human–nature bonds embody a connection to nature and existence in the world on different levels. These include experiential, based on personal experiences of individuals; contextual, or on a place-based level; planetary, relational to the Earth; and universal, the meta-Cosmos level. Human–nature bonds forming the web of life are in a constant and non-linear motion (Yellow Cloud and Redvers 2023). Indigenous People and local communities like farmers, fishermen, citizens, and reindeer herders relate to nature in different yet impactful ways stewarding sustainability. For instance, for many Indigenous Peoples human–nature bonds are relational and ontological. Betasamosake Simpson (2017) explains, “different human and nonhuman nations make up our world. They share and generate story, ceremony, song, learning, and action.” Watts (2013) argues that Indigenous understanding of “Place-Thought” demonstrates that agency is not limited to humans.

For many non-Indigenous farmers and fishermen human–nature bonds can be co-created and maintained through traditional land and water management practices (Plieninger et al. 2015; Hanaček and Rodríguez-Labajos 2018). Toledo and Barrera-Bassols (2009) explain the complex and systemic relationship between the environment and humans through history as “biocultural memory” expressed in thousands of philosophy systems, languages, and practices forming traditional knowledge. For urban and semi-urban citizens, human–nature bonds are rooted in resilient and sustainable environments and climate actions (Seymour et al. 2020). Besides colonialism, conflicts arise due to a lack of government accountability for environmental and social sustainability in favor of economic growth, neglecting local perspectives on extractivist projects (Joss 2010). In their book “We are Nature defending itself,” Fremeaux and Jordan (2021) argue that activists in the ZAD (‘zone to defend’) in Nantes, France, against an international airport infrastructure, displayed a diversity of tactics including day-to-day ritual practices in which people and non-human networks (trees, animals) collectively organized to live in and defend the ZAD. Human–nature bonds are preserved even when access to land, water, forests, parks, and similar have been affected by a project, taken away without consent, colonized and destroyed. In many instances people mobilize to reclaim historical memory in relation to human–nature bonds. The “Landback movement” (https://landback.org/) among Indigenous peoples in Australia, Canada, Native Americans in the United States is one example of this kind of grievance. As Yellow Cloud and Redvers (2023) put it, human–nature relationship is “everything that has ever existed and will ever exist.” Human–nature bonds, thus, are often *manifested* in peoples’ ancestral origins, customs, religions, spiritualities, storytelling, protection of history, mutual learning, respect for sacred sites, the responsibility people hold for nature, sustainable land-use and management practices, established autonomous zones or territories as well as economies (Kimmerer 2015; Magallanes 2015; McGregor et al. 2020; Rodríguez and Inturias 2018; Fremeaux and Jordan 2021). In this way, people develop, enhance, preserve, and defend human–nature relationships.

Temper and Martinez-Alier (2013) showed that environmental conflicts with social, environmental, and cultural impacts are not about negotiation for a *“better economic deal,”* but make visible different ways of how the environment is valued and protected. These understandings enable a holistic analysis in which human–nature bonds come to matter in environmental justice struggles (Guha and Martinez-Alier 1997), emphasizing thereby the role of human–nature links in reifying and resisting historical extractivist state-corporate coloniality and hegemonies (Baviskar 2007; Kojola and Pellow 2020; Sultana and Loftus 2012).

### Colonialism and extractivism playing against human–nature bonds

Colonialism is a historical process through which “one society is forcefully assuming control of another society’s territories and imposing its own systems of laws and governance, including natural resource management” (McGregor et al. 2020 p. 36). Often, the process entails neglecting political organizations for the sake of imposing the dominant model, usually Western, industrial, and extractivist (Acosta 2011; McGregor et al. 2020 via Whyte 2017a). The concept of (settler) colonialism explains the historical process of domination, slavery, and territorial invasion of Indigenous and African Peoples for trade and profit (Casanova 1965). Internal colonialism, however, refers to the same colonial logic maintained internally to a modern nation state by political powers who have historically benefited and profited from colonial world order, like the elite (Casanova 1965; Rivera Cusicanqui 2010; Fanon 1961). For example, in their work on nuclear colonialism in the US, Churchill and LaDuke (1992) argue that radioactive waste disposal continues to contaminate internally colonized areas and bodies judged inferior, resulting in what Fanon (1961) and Cabral (1973; English translation 2016) have called dehumanization, erasure of history and culture, and psychological destruction as continuous coloniality in a contemporary society. The term of internal colonialism, thus, explains a continuing colonialism into the present day, where colonized peoples and territories are incorporated (often by force) into colonized power (Churchill 2012). The settler colonialism also involves internal colonialism, however, with colonizing power not only meaning “overseas” (Churchill 2012; Etkind 2015). Examples include Siberia in today’s Russian Federation and Adivasi lands in India (Dey 2019; Etkind 2015; Rycroft 2011; Vakhtin 1992). Also, in the East, there were Japan’s relentless attempts at imperialism until 1945 (Caprio 2009). Another main thrust of colonialism from the sixteenth century onwards was imposing ideologies of *Terra nullius*, bringing along blindness towards other deep connections to and meanings of nature (Pilgrim and Pretty 2010; Willow 2016). Coloniality still tidily holds control, domination, and exploitation over lands, nature, and historically oppressed people, notably Indigenous, the racially discriminated, and women; if not the combination of the three axes of difference (Crenshaw 1991; Pulido 2017; Sultana 2020).

Meanwhile, extractivism is a power dynamic underlining socio-ecologically destructive modes of life through subjugation, violence, and depletion (Chagnon et al. 2022) causing vanishing connectedness and interdependence between people and nature (Acosta 2011; Svampa 2015; Willow 2016). As such, extractivism relies on epistemological and ontological processes of overlooking or denying most non-dominant existences and affects different ways of life across places in which peoples are subject to oppression (Kröger et al. 2021). Therefore, human–nature bonds as a resistance against colonialism and extractivism are an important point for examination, as the concept sheds light on the significant social consequences embedded in profound systemic and political inequities (Willow 2016).

Seeing nature only as an extractive resource not only favors growing and changing social metabolism as well as the unequal exchange of energy and material resources, but also neglects ways people create the relationship with nature (Lorde 1999; Martinez-Alier et al. 2010). Alimonda et al. (2011) and Whyte (2017a) explain how such environmental justice conflicts are, in fact, issues of a much larger story. According to the authors, colonialism through extractivism continues to work to erase Indigenous peoples culturally, economically, and politically. Alimonda et al. (2011) further argue that colonialism is not just about changes in society/nature interaction, but about profound power relations throughout history, including violence, that are mediated by extraction of natural resources even today. Willow (2016) asserts that extractivism disrupts communities’ capacity to function as effective independent entities. This is because extractivism causes violent displacement wherein they are not only harmed and/or assassinated in the process of removing them from disputed territories, but in doing so, extractivist projects remove communities from their means of supporting their livelihoods (beyond economic, this includes physical, spiritual, and emotional wellbeing), leading to impoverishment and famine (Tran and Hanaček 2023). Moreover, extractivism contributes to communities’ persecution and extermination whereby any survivors are assimilated and their culture erased (Crook and Short 2021; Lynch et al. 2021). This social–environmental aspect is what concerns us in the present article because it is the foundation of sustainability and justice (Scheidel et al. 2018).

### Resistance

Struggles against extractivism in defense of lands, mountains, landscapes, icescapes, forests, rivers, lakes, and the like involve pluri-ontological understandings of human–nature relationships (Escobar 2018, 2016, 2008; Jarratt-Snider and Nielsen 2020; Kagawa-Viviani et al. 2018; Klain et al. 2017). Although environmental conflicts are movements of ecological, social, and cultural meaning to a place locally, these form part of the anti-colonial and anti-extractivist resistance globally (Escobar 2001; Gilio-Whitaker 2020).

If we think of environmental conflicts as social conflicts over the environment (Le Billon 2015; Robbins 2003), then we think of peoples’ resistance to the destruction of human–nature bonds as an important segment of environmental conflicts (Hanaček and Rodríguez-Labajos 2018; John 2016). The literature has demonstrated that such conflicts, in most cases, cannot be solved merely by monetary compensation, because this simplifies complex ontological and epistemological components related to the environment and reduces them to extractivist ideologies (Gudynas 2009; Guha and Martinez-Alier 1997; Martinez-Alier 2002; Temper and Martinez-Alier 2013). This is because extractivist tendencies underlie unsustainable practices while simultaneously including ideologies based on economic models (Kallis et al. 2013; Kröger et al. 2021). Moreover, this agenda denies the legitimacy of different cultures, economies, religions, knowledge, and worldviews co-created *with* and *within* many environments (Escobar 2018; Hanaček et al. 2022; Jarratt-Snider and Nielsen 2020; John 2016; Martinez-Alier 2002; Munda 2008; Reyes-García, 2015).

Humans and nature are inherently linked (Cevasco et al. 2015; Díaz et al. 2018; Escobar 2008). This human–nature relationship is expressed in collective identities and values in environmental justice struggles (Escobar 2018; Pulido and De Lara 2018). Due to extractivist undertakings, however, these sites often become contested and thus highly politicized places (Breslow 2014; Kahanamoku et al. 2020; Willow 2016).

In truth, colonial politics, policies, and practices historically and presently work to reinforce the acculturation of and the erasure of non-dominant lifeways (Steeves 2018). An in-depth understanding of local struggles for nature is necessary not only for local protection of nature and conceptualizations of it, but also for recognizing local struggles against inequality and colonialism enacted throughout history (Ducarme and Couvet 2020; Hicken et al. 2021). Standing on the frontlines for transformative governance through struggles for liberation to the defense of world-views, human–nature bonds, and anti-colonial ecologies, economies and cultures, which has been neglected for more than five centuries should finally be recognized (Gobby et al. 2021; Rivera Cusicanqui 2010).

Current understandings of environmental processes deeply embedded within social justice and self-determination for people whose livelihoods are threatened by historical and contemporary colonial, capitalist, and extractive nexus are incomplete (McGregor et al. 2020; Muradian and Gómez-Baggethun 2021). Such views do not allow for moving beyond dualistic understandings of environmental sustainability (Escobar 2018). The overwhelmingly dualistic representation divides people from nature, and often involves so-called experts or other more powerful actors to decide where, how, and why nature should be either extracted or protected (Moola and Roth 2019; Pretty 2011).

The globalized economy, with its strongly extractivist character, destroys human–nature bonds, along with people being displaced from their lands and being subjected to poverty. Poverty, here, does not mean low income, but mainly deprivation of access to land, water, and clean air (Sullivan and Hickel 2023). Thus, these extractivist and colonial tendencies have sustained relations in which people are not only dispossessed of their means of maintaining economies, ontologies, epistemologies, and human–nature relationships, but also exposed to environmental degradation, toxicity, impoverishment, and food insecurity; which combined, sustain state-capital hegemonies worldwide (Tuhiwai Smith 1999). In this sense, ecological relational practices–a strong relationship between space, culture, and identity—is important for understanding struggles against the destruction of human–nature bonds (Escobar 2011; Gould et al. 2019; Waldron 2018).

## Methods

### Data sources: the Global Environmental Justice Atlas (EJAtlas)

The EJAtlas is a valuable database making diverse voices for environmental justice visible, as it provides examples of resistance to environmental destruction embedded in colonialism and capitalism worldwide. In the EJAtlas, human–nature bonds that retain land, water, human rights, heritage, and political standings are well documented and collected between grassroot organizations, civil society, and academics (Temper et al. 2015, 2018). Although the EJAtlas provides many examples in which social movements call for environmental justice (Jarratt-Snider and Nielsen 2020 p. 4; Martinez-Alier et al. 2016), the EJAtlas has some shortcomings. For instance, the database covers some geographical areas of environmental conflicts more than others due to the availability of data and local contacts (Scheidel et al. 2020). The EJAtlas is an archive of environmental conflicts, some of which ended some time ago. Other conflicts are still active, and require updating, consultation with local communities, and careful monitoring of secondary data, especially when court cases, forms of mobilization, and company investment advance and might change the outcomes of a conflict.

Nevertheless, the EJAtlas has proven to be an important database for studying comparative socio-environmental problems and theorizing about land defense (Le Billon and Lujala 2020; Scheidel et al. 2020), violence towards “women environmental defenders” (Tran 2021), surgency of commodity frontiers and colonialism (Hanaček et al. 2022), successful mobilization strategies against extractivism (Temper et al. 2020; Walter and Wagner 2021), and working-class communities and toxic pollution in environmental health conflicts (Navas et al. 2022).

Impacts on human–nature bonds were identified through an EJAtlas variable indicating the impact on traditional knowledge, practices, and cultures (Supplementary material). The variable is defined as “the loss, decline, or distortion of knowledge, practice, and beliefs maintained through generations, social cohesion, cultural transmission, the relation between humans, non-humans, and other-than-human natures.” Examples may include “loss, decline, or distortion of knowledge about animals, crops, plants and medicine, sacred places and meanings, sounds of forests, lands, rocks, mountains, and rivers; oral traditions such as storytelling, songs, and arts; spiritual and religious rituals, tribal laws, identities, and land managements,” which together indicate a long-preserved bond between humans and their environments.

However, limitations of this general definition do not capture deep social, cultural, and environmental meanings of a given place, such as sacredness. Therefore, we choose to focus our analysis on those *human–nature impacts* that are closely linked to “environment and politics, and nature and society” (Nygren and Rikoon 2008, p. 770). The following section provides descriptions taken from the EJAtlas of some emblematic human–nature impact cases under consideration before engaging in global statistical analysis of the data.

### Examples of human–nature bonds in environmental justice conflicts

One important example is Mauna Kea, where a $2.1 billion- and 30-m-high telescope would permanently alter the mountain, Hawaiian culture, and long-preserved human–nature bonds. Scholar and activist Case (2014) writes:

“Our ancestors have placed their faith in us that we will carry forth their mission to walk with careful steps on this land, in trust that we will not obstruct, destroy, or desecrate that which they held as most sacred. Just imagine what it takes to build something of that size, what will be carried up to the top of the mountain, and what will be left there when all is done. […] If you believe that something that immense will not create repercussions, I ask you to rethink that deeply. […] We have made too many consensuses, too many compromises. This time, we speak for the mountain. The mountain says no.”

In Australia, Tjiwarl people and women leaders have been opposing uranium mining in Yeelirrie proposed by a multinational uranium mining company whose intentions are to dig a 9-km-long pit over 2400 hectares of the Aboriginal lands (EJAtlas 2019a). As Tjiwarl People explain, the uranium mine would threaten important natural and cultural sites—part of the *Seven Sisters Dreaming Story Line,* which dates back 100,000 years ago. It is the world’s oldest story written across the night sky in the Kungakungaranga (the Pleiades star cluster) (Norris and Norris 2021). The Story Line is the foundation for Aboriginal culture and anchor of all sacred sites, women’s ceremonies and stories. The Story Line also defines a group of people across the territory, the land that they live on, and the law that they live under (see Neale 2017).

In Russia, mining at Kushtau hill in Southern Ural was successfully stopped after collective anti-mining actions (EJAtlas 2020a). As the hill has high social, cultural, and environmental value, local people call it a sacred hill. This is especially true for the Bashkir people, as the hill for them is a ritual site steeped in human–nature bonds. People explain that the hill protects the residents from big shocks and upheavals. Following the action, the site has been designated as a protected area.

In Norway, Fosen Vind, consisting of 151 turbines and owned by two government companies, is one of the biggest wind power projects in Europe (EJAtlas 2021a). However, the project is harming Sami people because it is built on their ancestral territories important for the survival of the reindeer herds. Following a disagreement on the project in November 2021, the country’s Supreme Court has ruled that “concessions for the wind farms are invalid and should never have been granted” and, therefore, withdrawn. More than 500 days after the Verdict, windmills have not been removed from Sami homeland. In February 2023, in a peaceful gathering in front of the Ministry of Petroleum and Energy headquarters in Oslo, Sami youth made an urgent request to the government of Norway to adhere to the decision of the Supreme Court (IPRI 2023). However, the demonstration was suppressed by the Norwegian police.

Meanwhile in Uganda, Indigenous Acholi women fought off land-grabbing for a game park. By performing cultural rituals including a “naked curse,” the women convinced local decision-makers to drop the project (EJAtlas 2021b). This culturally imbued symbolic meaning of their actions deeply moved investors and authorities. Likewise, Indigenous Sápara leader Gloria Hilda Ushigua Santi fought against the Amazonian oil industry destroying their already endangered ancestral heritage (EJAtlas 2020b). She organized protests wherein people wore traditional clothing, sang, and performed rituals. Thus, in many conflicts, land protectors politicize their cultures, traditional knowledge, and practices through various means to counter extractivist hegemonies.

### Data analysis: descriptive statistics and log-linear regression

We examine a sample of 1882 cases indicating impacts on human–nature bonds, presenting similar features as those described in “Examples of human–nature bonds in environmental justice conflicts”. Here, we apply statistical log-linear regression. The regression analysis has been widely used to examine categorical, non-linear, and non-mutually exclusive variables of socio-environmental dynamics (see Bijl et al. 2017; Dietz and Rosa 1997; Loreau and Hector 2001; Screen and Williamson 2017). The analysis is suitable for data from the EJAtlas, which contains more than one response of categorical variables and registers diverse socio-ecological impacts globally (Navas et al. 2022; Scheidel et al. 2020; Temper et al. 2018).

Identified categories indicating impact on traditional knowledge, practices, and cultures of the EJAtlas were coded in 0 or 1 format in combination with the following categorical variables: conflict type (e.g., nuclear, waste management), commodities (e.g., coal, oil, land), impacted social groups (e.g., farmers and peasants, Indigenous peoples), forms of mobilizations (e.g., street protests, official complaint letters, media-based activism), and conflict outcome (e.g., achieved alternative solution, court case decision in favor of groups protesting (or not), assassination of activists or project canceled).

The first step was to select all conflicts in the Atlas that report impacts on traditional knowledge, practices, and cultures (see definition in “Data sources: the Global Environmental Justice Atlas (EJAtlas)” section) to obtain a systematization of visible (*n* = 899) and potential (*n* = 983) impact on human–nature bonds (total *n* = 1882, 53%) while at the same time looking at conflicts that do not report that impact (*n* = 1649, 47%). Potential impact refers to all those conflicts that mention the impact when the project is in its early stages or the exploratory phase (proposed) or to contested projects that have been stopped, postponed, canceled, and, therefore, no longer have the impact. In fact, 58.7% of cases reporting the loss show preventive resistance, while about 42% indicate a reactive mobilization stage.

The second step was to apply descriptive statistics and log-linear regression (Imrey et al. 1981) for impacts on human–nature bonds with each of the above-mentioned variables separately, because not all the variables are mutually exclusive. Log-linear regression is a useful analysis for frequency and associations between data and predicts the number of observations between them, which results in a probability of their occurrence (Freund et al. 2010; Imrey et al. 1981). We tested whether there are statistically significant differences across the variables indicating impacts on human–nature bonds, and those that do not indicate the impact in the database. We follow Navas et al.’s (2022) procedure, whose study included those cases reporting health impacts versus those cases that do not report health impacts. This approach allowed us to systematically synthesize and theorize environmental conflicts related to impacts on human–nature bonds.

The following results section shows only those variables that showed statistically significant differences (*p* ≤ 0.05) of co-occurrence with the impact or without such impact, including positive and negative relationships. If a *relationship* (*r*) is positive, it means that as one variable increases (e.g., biomass and land conflict), the other (human–nature impact) tends to increase. If *r* is negative, then it means one variable increases (e.g., waste management), while human–nature bond impact tends to decrease. Here we considered a statistically significant difference to be with a margin error of ≤ 10%. While a statistically significant difference ≤ 0.05 indicates that there is a 95% or more probability of the co-occurrence within the observed sample, the margin of error indicates the percent (%) of difference from the actual sample. This means that not all phenomena can be explained merely by statistics and that there is uncertainty with data sources and the statistical analysis itself. According to the margin of error, the estimation of the uncertainty in this article is not higher than 10% (Supplementary material).

Statistical political ecology analysis was used for three main reasons. The first reason is that the EJAtlas database includes conflicts other than human–nature bond conflicts (such as public health hazards from industrial toxins), therefore we employed log-linear statistics to determine the accuracy of the subsample in terms of conflict type and protagonists involved in such conflicts. The second reason is that our analysis is conducted on a global scale; therefore, differences in location and conflict outcome require a statistical comparison approach. For instance, refusing economic compensation for damages in human–nature bond conflicts in comparison with cases that do not indicate impact on human–nature bonds. The third rationale for statistical political ecology methodologies is that the approach extends beyond a particular case study (Sheidel et al. 2023; Tran and Hanaček 2023), demonstrating how extractivist and colonial connections influence global ecologies through human–nature relationship expressions.

## Results

### Drivers of change across cases reporting impacts on human–nature bonds and affected social groups

**Fig. 1:**
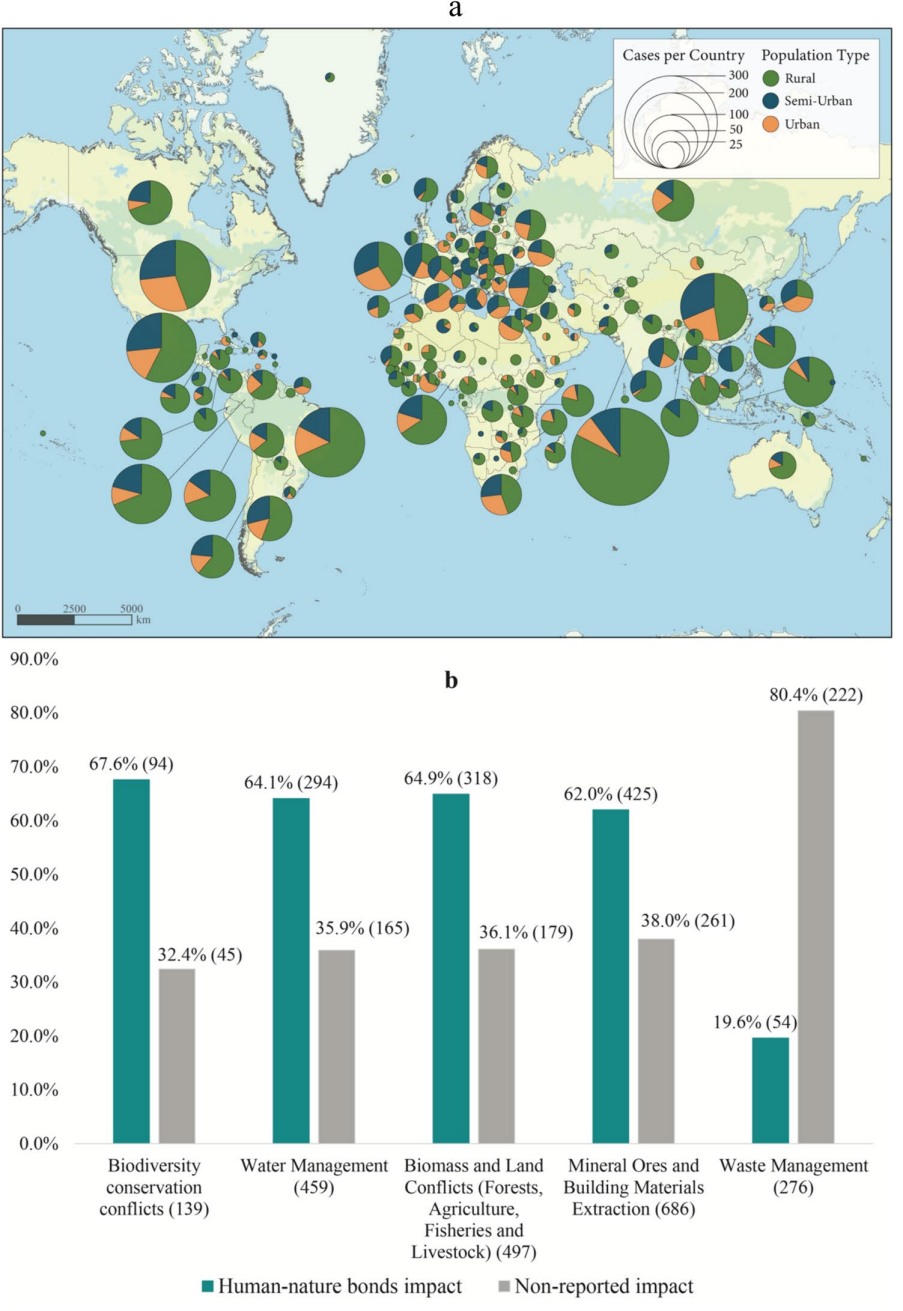
Global distribution of cases indicating impact on human–nature bonds across rural, semi-urban, and urban areas summarized by each country from 1860 (as the earliest start of a conflict identified in the database) onwards (**a**); Conflict type indicating impacts on human–nature bonds (**b**). Variables are mutually exclusive. Visible and potential human–nature bonds impact reported (green) within the cases = 1882. Non-reported human–nature bond impact (grey) within the cases = 1649. Total observed cases = 3531. Only statistically significant results (*p* ≤ 0.05) with an error margin of up to 10% are included. For the detailed relationship between the categorical variables, see log-linear regression in Supplementary material

Figure 1a shows the distribution of cases with impacts on human–nature bonds across rural, semi-urban, and urban areas globally and summarized by each country. According to the regression analysis, 67.6% of biodiversity conservation conflicts, 64.1% of water management conflicts, 64.9% of biomass, and land conflicts, and 62% of mining conflicts show a positive relationship and statistical significance (*p* < 0.05; error margin 1.68–5.34) with impacts on human–nature bonds in comparison with cases that do not report such impact (Fig. 1b). For example, the Xingu Basin in Pará and Mato Grosso (Brazil) has always been subject to environmental depletion and violation of Indigenous rights (EJAtlas 2021c). Indeed, land inequality has become widespread in Brazil since colonial times owing to powerful elites openly using violence and systematic assassinations to deter community resistance to extractive projects (Barbosa and Roriz 2021; Tran and Hanaček 2023). However, from 2018 onwards, the agribusiness sector’s violent encroachment upon Indigenous lands has increased in intensity and scale with support from Jair Bolsonaro’s authoritarian populist government. Main factors in increase of environmental conflicts in the Xingu basin are the expansion of soya plantations and the effects of Belo Monte dam. The violence follows patterns of repression wherein dictatorships embolden impunity for violent land-grabbing amidst strong economic incentives to exploit land and natural resources, marginalization of those dependent on such resources, and rampant corruption (Tran and Hanaček 2023). Such an uptick in extractive displacement has been captured by NGOs’ satellite monitoring and reporting (EJAtlas 2021c).

Furthermore, a proposed pipeline from the Russian Federation to China across Tunka National Park next to Lake Baikal was successfully stopped by local people. Values of respect for and sacredness of nature were explicitly stated by land protectors, including mountain spirits and places of worship (EJAtlas. 2022). Across waste management conflicts, however, we observe a negative relationship with impact on human–nature bonds (19.6%; *p* < 0.05; error margin 10%), suggesting that in such conflicts, human–nature bonds are not frequently expressed. Such conflicts instead emphasized health impacts, work insecurity, and wages (Navas et al. 2022).

**Fig. 2:**
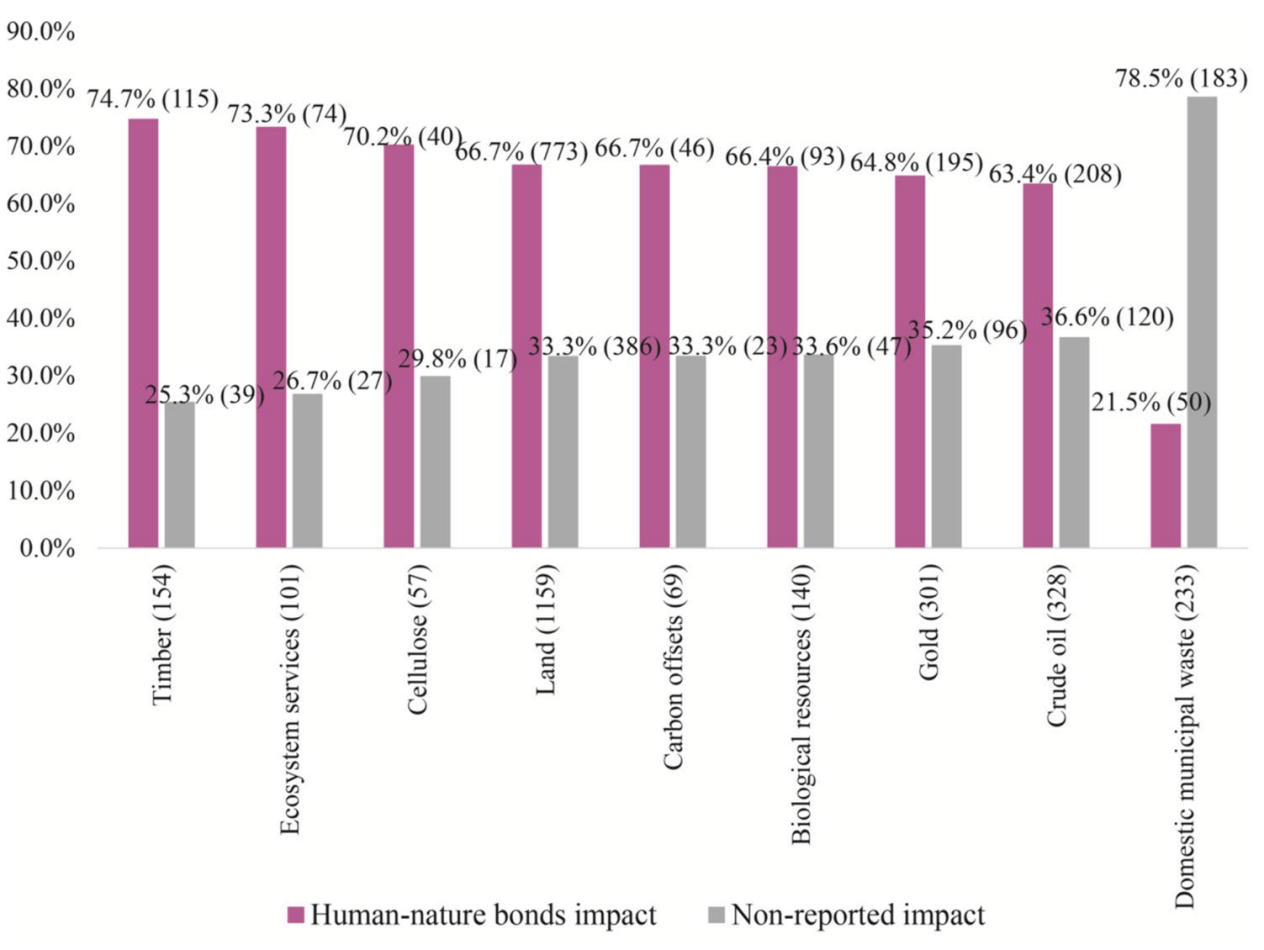
Commodity extraction related to impacts on human–nature bonds. Variables are *not* mutually exclusive. Visible and potential impact on human–nature bonds reported within the cases (pink) = 1882. Non-reported human–nature bond impacts (grey) within the cases = 1649. Total observed cases = 3531. Only statistically significant results (*p* ≤ 0.05) with an error margin of up to 10% are included. For a detailed relationship between the categorical variables, see log-linear regression in Supplementary material

Moreover, those cases involving intensive agriculture and forestry-based commodities, such as timber (74.7%), cellulose (70.2%), and land (66.7%) specify impacts on human–nature bonds in comparison with those cases that do not report the impact (*p* < 0.05; error margin 2.42–9.75) (Fig. 2). Then, cases involving commodities related to mining and oil extraction, such as gold (64.8%) and crude oil (63.4%) again indicate human–nature bond impacts (*p* < 0.05; error margin 2.54–3.0%).

Similarly, cases where carbon offsetting and ecosystem services have been monetized suggest highly significant impacts on human–nature bonds (66.7% to 73.3%; *p* < 0.05; error margin 6.94–8.94). For example, when receiving resources from the carbon credit sales from the “Carbono Suruí” project, in Cacoal, Rondônia (Brazil) leaders of the Suruí people expressed their concerns, stating that, “there has been no transparency about the use of the money, families have not been granted better living conditions and local economic activities are particularly affected by the monetarized project.” Suruí peoples could not continue their land use, including hunting (EJAtlas 2019b).

Overall, a negative relationship with human–nature bond impacts is found in cases with commodities such as domestic and municipal waste (21.5%; *p* < 0.05), suggesting, again, more urban conflicts related to impacts on health, work insecurity, and livelihood loss of informal recyclers (EJAtlas 2019c). However, such cases can indicate that human–nature bonds became a mobilized discourse (error margin 9.94). For example, the Muthurajawela wetlands are the largest saline coastal peat bog in Sri Lanka (EJAtlas 2021d). However, the place has been used as a solid waste deposit. Local fisherman’s livelihoods, fishing practices, and health are highly affected by this waste dump.

**Fig. 3:**
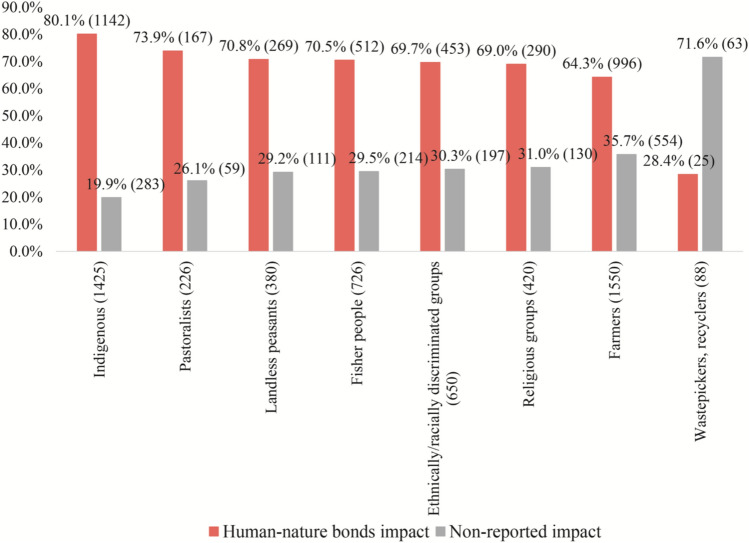
Affected groups across cases related to impacts on human–nature bonds. Variables are *not* mutually exclusive. Visible and potential impacts reported (red) within the cases = 1882. Non-reported impact (grey) within the cases = 1649. Total observed cases = 3531. Only statistically significant results (*p* ≤ 0.05) with an error margin of up to 10% are included. For a detailed relationship between the categorical variables, see log-linear regression in Supplementary material

Affected social actors that mobilize in environmental conflicts with reported impact on human–nature bonds (positive relationship, *p* < 0.05; error margin 2.03–6.42) are Indigenous Peoples (80.1%) and ethnically or racially discriminated groups (69.7%) (Fig. 3). These impacts also occur at the expense of pastoralists (73.9%), landless peasants (70.8%), fisher people (70.8%), and farmers (64.3%). Furthermore, institutionalized, and professional groups such as religious groups appear in 69% of conflicts with a positive relationship (*p* < 0.05) across cases (Fig. 5). For example, copper mining activities by a Chinese company near Lingka Monastery in Tamo (Tibet) was interrupted by Tibetan monks and the general public (EJAtlas 2018). There have been many protests in Tibet against environmental destruction, such as mining in and near sacred mountains and holy lakes. However, many of the protests have been violently suppressed by Chinese authorities (CTA 2015).

In addition, Afro-Colombian, Indigenous, women’s, peasants, and environmental collectives have been dutifully defending the “relational weave of life” with their territories (Escobar 2018). In 2006, for example, these collectives gathered in a preventive mobilization against gold mining in Dojura, Chocó region (Colombia) (EJAtlas 2014). Mobilizers expressed concerns about mining by multinational companies, displacement of local communities and/or assassination of activists, as well as armed groups and paramilitaries dispatched by corporations and governments to enact such violence and displacement. The collectives indicated that such criminal activities would also erase historically, culturally, and spiritually important Afro-descendant territories. Thus, history and collective memory shape environmental justice struggles.

### Forms of mobilization and conflict outcomes across cases reporting impacts on human–nature bonds

**Fig. 4:**
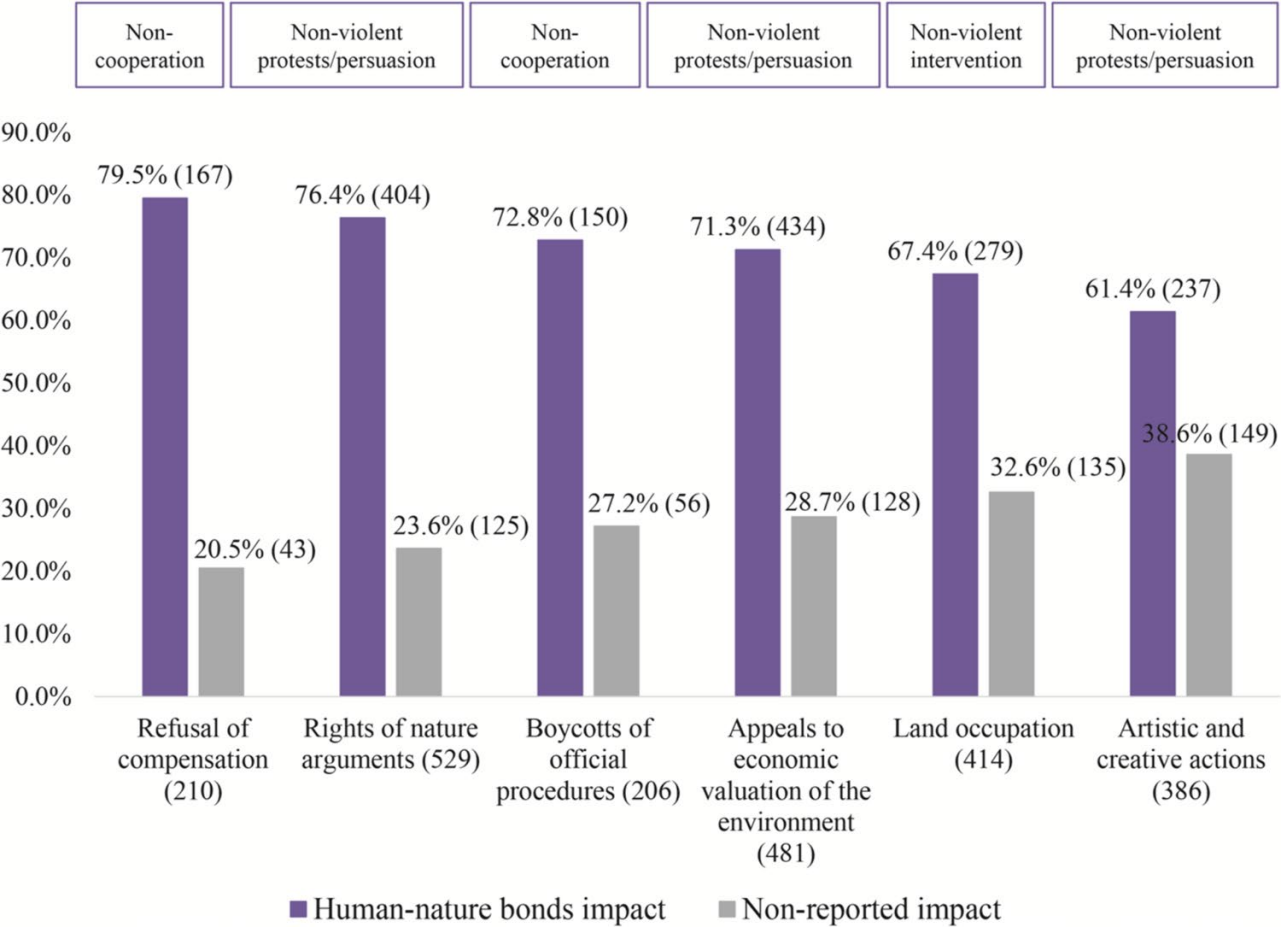
Forms of mobilization (grouped as in Scheidel et al (2020) via Sharp (1973)) across cases reporting impacts on human–nature bonds. Variables are *not* mutually exclusive. Visible and potential loss reported (purple) within the cases = 1882. Non-reported impact (grey) within the cases = 1649. Total observed cases = 3531. Only statistically significant results (*p* ≤ 0.05) with error margin up to 10% are included. For a detailed relationship between the categorical variables, see log-linear regression in Supplementary material

In terms of non-violent protests across the subsample (Fig. 4), there is a positive relationship between rights of nature arguments and impact on human–nature bonds (76.4%; *p* < 0.05; error margin 5.25) in comparison with cases that do not indicate the impact. In the Hawaiian case mentioned in “Examples of human–nature bonds in environmental justice conflicts”, for example, the rights of nature were strongly expressed through cultures, system beliefs, and world views by environmental protectors who hold strong connections with the mountain, the water source on the mountain, and even their relationship with outer space*.* In addition, in their court filing against the Dakota Access Pipeline route under the Missouri River, US, the people of Standing Rock stated “Our primary concern is water […] we are here to pray, and we will continue doing it. Those prayers come from a deep understanding of a relationship with Mother Earth and offerings are made to Her as appeals are made.” (Native Knowledge 2016; EJAtlas 2016; Alkire n.d.). As noted, environmental conflicts indicating impacts on human–nature bonds reveal specific cultural expressions as a form of resistance with an obligation and responsibility to care for the bonds (LaDuke 2016).

Concerning non-violent interventions, cases reporting the impact have a positive relationship with forms of mobilization such as land occupation (67.4%, *p* < 0.05; margin error 3.29) in comparison to the cases that do not report human–nature bonds impact. There are many examples of land occupation as a form of mobilization. For instance, Navajo Native American peoples highly disagreed with the artificial wastewater snow project at the San Francisco Peaks; explaining that the project entails huge negative consequences for mutually interrelated socio-environmental systems, including the weather, the water, their culture and religious practices, the land, and the life on the land (EJAtlas 2021e).

There are many ways in which threatened peoples mobilize their human–nature bonds and ways of life in their resistance. For example, Afrida Erna Ngato in North Maluku, Indonesia, restored a traditional women’s leadership role and customary governance style that previously went culturally extinct (EJAtlas 2021e). The villagers documented their history and culture and mapped tribal borders, effectively mobilizing their Indigenous knowledge as evidence bolstering litigation, standing up for their rights, and making it more difficult in the future for mining companies to exploit them. Iñupiat tribal leader Caroline Cannon likewise mobilized traditional knowledge of the Arctic marine environment in Alaska as compelling evidence in court to successfully pressure the US government into canceling fracking leases (EJAtlas 2020e).

Explicit refusal of economic compensation is rarely found in the EJAtlas. Often, non-economic valuation languages are deployed but there is no refusal of monetary compensation. In those cases that there is, we observe the prevalence of an impact on human–nature bonds in 79.5% (*p* < 0.05; error margin 8.91). Refusal of compensation, for example, was found in a contentious case of mining in the Pilbara region of Western Australia (EJAtlas 2021f). To mine iron ore, the Rio Tinto company has blown up 46,000 years old Juukan Gorge caves with explosives, sacred to Puutu Kunti Kurrama and Pinikura people. Although the companies have recognized the damage, have publicly apologized to the peoples, and could end up paying for restitution; the amount, however, is designated to raise awareness and education about Indigenous culture and heritage (Burton 2020).

Remarkably, appeals to economic valuation of nature appear across 71.3% of cases (*p* < 0.05; error margin 4.07). The following case of deep-sea oil drilling in New Zealand exemplifies the monetary dispute in the process of resistance with a potential impact on human–nature bonds (EJAtlas 2019d). A Norwegian oil company has an agreement with the British Crown to drill for deep-sea oil off the coast of Ahipara. The company and the Crown, however, did not consult about the project with the Māori people, who have rights over the area and who strongly oppose exploration, seismic survey, or drilling of the seabed and land itself. The Māori people stated that if an oil spill happens, “the Government requires companies only to pay a small amount towards cleaning up the spill, while the cost to peoples’ livelihoods and economy is devastating, requiring billions of dollars in restitutions.” This case also relates to refusal of monetary compensation, as the Māori people state that it is unlikely compensation could ever make up for an oil spill on their ancestral territories.

**Fig. 5:**
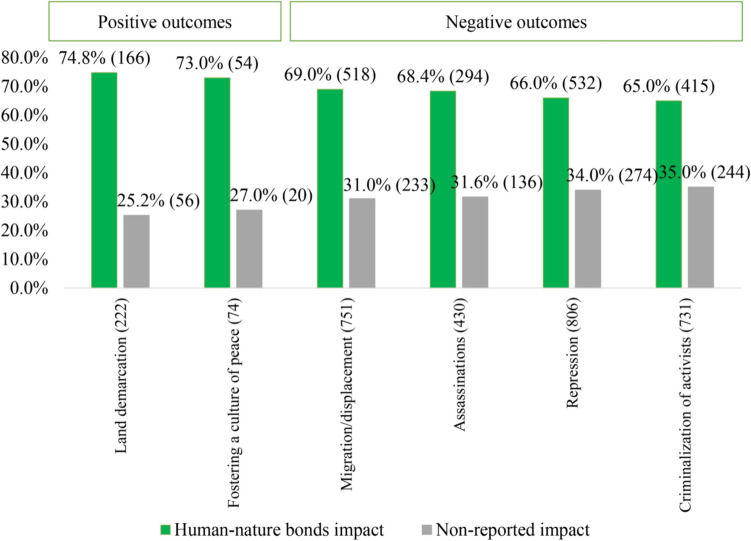
Conflict outcomes in cases with human–nature bond impacts. Variables are *not* mutually exclusive. Visible and potential impact reported (green) within the cases = 1882. Non-reported impact (gray) within the cases = 1649. Total observed cases = 3531. Only statistically significant results (*p* ≤ 0.05) related to different conflict outcomes are included with error margin up to 10%. Outcomes are grouped in positive and negative outcomes as in Scheidel et al (2020). For a detailed relationship between the categorical variables, see log-linear regression in Supplementary material

In those conflict outcomes with land demarcation, 74.8% specify impacts on human–nature bonds (*p* < 0.05; error margin 6.79). In addition, 73% of outcomes with fostering culture of peace indicate impacts on human–nature bonds (*p* < 0.05; error margin 10.22). Moreover, in cases where migration or displacement of communities take place, assassination of people protesting, repression, criminalization of activists, and violent targeting of activists occurred, we also found impact on human–nature bonds in 63–69% (*p* < 0.05; error margin 2, 0.25–3.05) (Fig. 5).

For example, Raspadskaya coal mining complex is located near Mezhdurechensk town in Kemerovo region (West Siberia), home to Indigenous Shor peoples (EJAtlas 2020c). For many years, Shor activists and their supporters have been opposing coal mining in the region. One of the activists was attacked twice, in 2013 and 2014. According to the activist, it is very likely that the attacks were part of the coal business intimidation plan as it controls armed checkpoints at the entrance to the village. Other intimidation tactics included a campaign organized against Shor activists on regional and federal levels. Despite this, the activists continued to express their concerns about coal mining that destroys peoples’ territories, Karagay-Lyash sacred mountain, ancestral burial grounds, and livelihoods.

In addition, an activist was officially informed by the police about criminal charges for organizing and participating in protests. The activist also lost her job at the school where she worked as a teacher and received multiple threats connected to her protest activity from representatives of the police and coal business. After several years of such pressure, the activist and her family left Russia and applied for political asylum elsewhere. Communities, activists, and journalists who stayed are still subjected to forced displacement and criminalization.

The same story repeated itself in Khakassia (South Siberia) (EJAtlas 2020d). Several Indigenous villages have literally vanished due to coal mining. Activists inform about the worrying situation on the ground: “Have you seen the dust? This is solid dust, solid dust. Where to escape from dust? Nowhere. Explosions! […] Water is disappearing. All this is coal dust”. The extensive coal mining also affects the Khasas people’s sacred water spring, Ymajrykh, considered to be a womb of Mother Earth and greatness Goodness Ymaj, meaning The Mother of Khasas land. Pure water from springs is essential for water ceremonies upon which cultures, worldviews, and life are based.

## Discussion and conclusion

How human–nature bonds are protected against extractivism is an important angle in environmental conflict research and sustainability science (Adams 2010; Breslow 2014). The EJAtlas reports environmental conflicts globally, many of which include impacts on human–nature bonds (1882 cases or 53% of the database). This article has sought to understand such impacts. Based on log-linear regression, the obtained results suggest that environmental conflicts related to, for example, access to land and water (Peluso and Lund 2011; Ribot and Peluso 2003), develop in tandem with negative impacts of extractivism on human–nature bonds. Colonialism negates Indigenous legal orders, knowledge, principles, and values related to nature (McGregor et al. 2020). The same observation applies to biodiversity conservation initiatives. When such initiatives are developed under an extractivist and colonial logic, deciding and selecting what kinds of life are desirable, in what configuration and arrangements and where, this also leads to changes in culturally important landscapes, ecosystems, and human–nature relations (Mora-Motta et al. 2020).

Environmental conflict struggles are processes where marginality is deconstructed within spaces of resistance that reclaim social justice (Lorde 1999). By taking into consideration human–nature bonds, this article suggests that nature is both historically and contemporarily maintained by the people (Escobar 1996) and, therefore, protected. Such protection is often expressed in but not limited to impacts on human–nature bonds. Life is made possible by reclamation of knowledge, practices, and cultures co-created with nature (Escobar 2018). As such, struggles can be understood as both the argument and a form of resistance (Watts 2013). That what has been systematically neglected and oppressed—peoples and nature relationships—is at the same time contested and used as resistance during environmental conflicts (Hanaček and Rodríguez-Labajos 2018). Affected people protect their connectedness to the nature tied to their territory by (re)occupying the land.

Protecting the environment through human–nature relationships during conflicts is, on the one hand, contested because of how extractivist destruction of lands threatens communities’ grounded ontologies and their very sense of being (Kröger et al. 2021). This becomes a key point motivating people to mobilize against state-corporate extractivist and colonial relations (Martins 2022). On the other hand, maintaining human–nature bonds is also a form of resistance because it counters extractivist-dominating logics by refusing to allow such ways of life, doing, and thinking (Willow 2016). As much as land acquisition may represent colonial relations embedded in extractivism, land occupation as a form of mobilization represents interventions resulting in a process where affected people ensure culturally, socially, and environmentally significant sites against destruction, and not only access to land and water resources. This is because people strongly relate to nature and form part of it.

Furthermore, appeals to economic valuation of the environment indicate, to some extent, power relations in negotiation processes over the environment and against erasure of human–nature bonds. Yet there is strategic mobilization against corporations, investors, and the state, who impose their views upon colonized peoples and spaces (Gustafsson and Schilling-Vacaflor 2021; MacLeod and Park 2011). Human–nature bonds have been maintained and protected within local environments and through the peoples’ economic, environmental, epistemological, and ontological significance as well. Thus, there are many environmental justice struggles without an exclusive appeal to notions of monetary compensations (Kallis et al. 2013; Munda 2008). In fact, monetary languages, or use of compensations once impacts have been felt, do not mean that affected people value nature in monetary terms. Rather, it means that in environmental conflict processes, such languages sometimes appear as a last resort in order to protect what is left of nature.

In this regard, Indigenous territorial rights, human rights, and human–nature bonds, through sacredness, knowledge keeping, and ceremonies related to ancestry, are barely considered in top-down environmental and economic decision-making without being previously translated into monetized language (Martinez-Alier 2002). This argument is also sustained by a relatively high frequency of compensation refusals, because human–nature bond aspects in environmental conflicts include the self-determination of a community and the common life (Chan et al. 2016; Escobar 2018; Gould et al. 2019; Muraca 2016). In cases under consideration, we cannot always separate economic dimensions in protecting nature, but certainly, monetary valuation became highly questioned in such environmental conflicts (Martinez-Alier et al. 2010).

Moreover, in conflicts where assassinations, migration and displacement, repression, and criminalization of environmental protectors have occurred, we found reported impacts on human–nature bonds as well (65–69%). Similar results were found in the study of Scheidel et al (2020), indicating cases as increasingly violent towards protectors and their intersectional vulnerability. We also found land occupation (67.4%) as a physical human barrier in the process of opposing a project on sacred lands, for example. However, such physical barrier mobilizations are often expressed through discourse, symbols, bodies, and rituals enacted within religious, political, economic, and social dimensions interwoven in environmental struggles (Vaughan 1999). These discourses and symbols, furthermore, articulate and challenge unequal power relations and identities sustained within the larger story of struggle, namely, colonialism and extractivism (Alimonda et al. 2011; Whyte 2017b).

For instance, each land taken over by extractivism reinforces colonial development models and cultural hegemonies based on continuous violence over peoples and territories (Nixon 2011). In fact, McGregor et al. (2020) indicates how violence is experienced immediately when non-Western philosophies, ontologies, and epistemologies are undermined. As such, extractivism becomes violent towards identities and the ways people relate to nature as well (68.4% of the reported impacts) (Fernández-Llamazares et al. 2021). These arguments are also confirmed because of other frequent appearances of forms of mobilizations as non-cooperation (refusal of compensations in 79.5% of the cases), rights of nature arguments (74.6%), and artistic and creative actions (61.4%). Similarly, across conflict outcomes fostering a culture of peace and land demarcation (73% and 74%, respectively) are frequently reported. That said, in face of the high rate of forced migration, displacement, and death of environmental protectors, the peoples reclaim and enhance the sovereignty of the identities and cultures dependent on territories threatened by colonial extractivism.

Our results align with previous studies on the intensity of a conflict, which indicate that besides overuse of resources, pollution, and impoverishment of a space, human–nature relationships and cultural-specific protection of the environment can change dynamics of the conflict (Baechler 1998). Environmental conflicts may intensify if, for example, migration and displacement are a parallel outcome of such conflicts (69%) (Baechler 1998; Bernauer et al. 2012). Still, violent dynamics towards environmental protectors highly depend on socio-political or structural factors (Baechler 1998; Butt et al. 2019; Le Billon and Lujala 2020), not merely on forms of mobilization such as physical barriers protecting lands.

Mobilizations based on a physical barrier of land not only prevent the material advance of extractive transformations of land but also defend the cultural meanings, practices, knowledge, and identities through the physical presence of resistance bodies and their system’s beliefs. Thus, this counter-hegemonic presence shifts the material and symbolic power of extractivism and creates a plural narrative about nature and its meanings (Escobar 2004). Artistic and creative actions, as another popular way of protest in conflicts affecting human–nature bonds, were complementary to land occupations of the analyzed conflicts.

During these physical occupations, the people use artistic expressions, such as banners, dances, and chants that not only respond to the social and political strategies of socio-environmental movement (Sanz and Rodríguez-Labajos 2022), but also form political practices that re-configure the disputed territory through actions of resistance and human–nature existence (Bourriaud 2002; Serafini 2018; Fremeaux and Jordan 2021).

Human–nature bonds are key to understanding environmental justice struggles (Adams 2010; Hanaček and Rodríguez-Labajos 2018) for people who strongly oppose global extractivism of nature, cultures, identities, ancestral relationships, sacred lands, knowledge and way of life. In the process, those protecting human–nature bonds do not typically negotiate nor seek monetary compensation for extractive consequences. Rather, people challenge often colonial discourses and extractivism by physically protecting their territories and resistance through cultural and artistic expressions, protests, and seeking justice. Although this is a powerful tool in peoples’ repertoires, there are also certain deep oppressive contexts wherein counter-hegemonies and actions instead make people targets for further violence, such as displacement, criminalization, oppression, and assassination. Such observations have also been noted in the work of Kröger (2022) and Tran et al. (2020).

In this article, we have argued that human nature bonds are linked to a wide range of global struggles and mobilization tactics for protecting human–nature relationships (Alimonda et al. 2011; Whyte 2017a, b). As such, human–nature bonds in environmental conflicts are inseparable from land defense (Watts 2013). Such bonds are part of anti-extractivist, anti-colonial, undemocratic ecologies and power structures that have long sustained destruction of human–nature life on Earth and beyond (Lynch et al. 2021; Tran and Hanaček 2023; Dunlap 2021; Fremeaux and Jordan 2021; Churchill and LaDuke 1992). Struggles involving human–nature bonds go beyond mere sustainable land management, nature conservation and preservation. Rather, human–nature bond actions are embedded in the defense of life as an anti-colonial movement. With this global analysis of human–nature bonds impact, we contribute to the literature highlighting such impacts as a consequence of global extractivism that protectors (not protestors) struggle against during environmental conflicts in defense of human–nature and other-than-human life through philosophies, histories, ontologies, epistemologies, and tactics grounded in anti-colonial sustainability, social justice, and existence (Case 2014; Escobar 2018; McGregor et al. 2020).

## Supplementary Information

Below is the link to the electronic supplementary material.

Supplementary file1 (DOCX 324 KB)

## Data Availability

All data are publicly available on the EJAtlas database (ejatlas.org).
