# Supplementary material for: “We are protectors, not protestors”: global impacts of extractivism on human–nature bonds
Source: Sustain Sci. 2024 Aug 23;19(6):1789–808. doi: 10.1007/s11625-024-01526-1 (PMC11543721; doi:10.1007/s11625-024-01526-1)
Supplement: Supplementary file 1 — Supplementary file1 (DOCX 324 KB) [file 11625_2024_1526_MOESM1_ESM.docx]

**Definitions of variables used in this articles are based on (Scheidel et al., 2020).**

| **Socio-economic impacts** | |
| --- | --- |
| Loss of traditional knowledge, culture, practices | The loss, decline, or distortion of knowledge, practice, and beliefs maintained through generations, cultural transmission, the relation between humans, non-humans, and more-than-human natures. Examples include knowledge about animals, crops, plants and medicine, sacred meanings and sounds of forests, lands, rocks, mountains, rivers; oral traditions such as storytelling, songs, and arts, spiritual and religious rituals, tribal laws, and identities. |
| ﻿ **Main conflict category**  **(1st level classification)** | |
| ﻿Biodiversity conservation conflicts | ﻿Conflicts involving terrestrial and aquatic nature conservation initiatives. Examples: natural parks and other conservation zones, REDD+, wildlife corridors, etc. |
| ﻿Water management | ﻿Conflicts linked to the access to and control over (mainly fresh) water resources. Examples include dams, transboundary water conflicts, inter-basin water transfers, desalination plants, water access rights and entitlements, water privatization, water treatment and access to sewage facilities, etc. |
| ﻿Biomass and land conflicts (Forests, agriculture, fisheries and livestock management) | ﻿Conflicts involving land uses for agriculture, forestry, livestock, or fisheries. Examples: land acquisition for agribusiness, tree plantations, livestock farms, aquaculture, timber logging, extraction of non-timber products, etc. |
| ﻿Mineral ores and building materials extraction | ﻿Conflicts involving mining, including extraction, transportation, waste material disposal, raw processing. Examples: conflicts linked to mineral ore exploration and processing, tailings from mines, extraction of building materials such as sand, gravel, quarries, etc. |
| ﻿Waste management | ﻿Conflicts involving waste management, either creating unequal environmental burdens and/or health problems in specific areas, or over privatization of waste. Examples include incinerators, co-incineration facilities, toxic and e-waste disposal, polluting landfills, uncontrolled or ﻿unwanted dumping sites, ship-breaking yards, waste privatization, excluding waste pickers from their recycling practices etc. |
| ﻿ **Mobilization forms (actions)** | |
| ﻿Refusal of compensation | ﻿Rejection of the form or criteria for assessing reparations, or directly rejecting money offered as compensation. |
| Rights of nature mobilization | ﻿Mobilization arguing that nature is not merely a property to be owned, but rather an entity which has an independent right to exist and flourish. |
| ﻿Boycotts of official procedures | ﻿Intentional non-cooperation and non-participation in official procedures, such as public hearings or official consultations. |
| ﻿Appeals to economic valuation | Arguments and methods valuing nature in economic terms as a means to defend against its destruction. |
| ﻿Land occupation | ﻿Physically occupy an area of land for an extended period, frequently sleeping and living there. Such occupations usually take place on the site of a conflictive project or contested land, to physically prevent the project from taking off and/or continuing while engaging in forms of alternative living and collectivity building. |
| ﻿Artistic and creative actions | ﻿Use of creative ways or art to draw attention to environmental conflicts. It can range from guerilla theatre, street plays, fairs and parties, music, puppet shows to murals, graffiti, banners, etc. |
| ﻿ **Outcomes** | |
| Land demarcation | ﻿Demarcation of lands is the formal process of identifying the actual locations and boundaries of Indigenous lands or territories or more broadly of clarifying territorial boundaries. |
| ﻿Fostering a culture of peace | ﻿Despite the conflict, communities and mobilizing groups have worked for peaceful resistance and for fostering a culture of peace. |
| ﻿Migration/displacement | ﻿Forced or otherwise induced movement of peoples due to the conflictive project or activity. It includes displacement according to resettlement programs or without any such scheme. It can be a direct impact of the conflictive project or an indirect, gradual consequence of it across time. |
| Assassinations | ﻿Death of one or more protestors, intentionally caused by a third party. Death can occur on the spot, for example when shooting to death environmental defenders, or be caused following wounds, rapes, tortures, etc. |
| Repression | ﻿Threat to subdue or act of subduing protests by institutional or physical force. Includes a variety of tactics (frequently including violent and coercive actions, violating rights) taken by government, or security staff, militias or corporate actors, to quell dissent and protests. |
| ﻿Criminalization of activists | ﻿It includes criminal prosecutions of individuals and abuses of civil and human rights, the opening of criminal investigations unlikely to reach the trial stage used to disarticulate, demoralize and discourage social protest, and the use of disproportionate sentences for offenses to punish practices often deployed in social protests. |

**Case entry example from EJAtlas**

**Title:** Oak Flat copper and molybdenum mining, USA


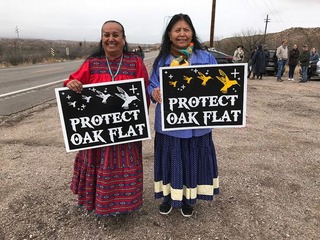
 Source: <https://justseeds.org/solidarity-with-oak-flat/>


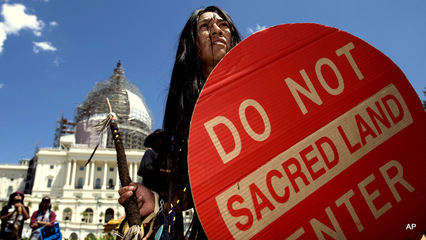


Source: <https://www.mintpressnews.com/arizona-apache-continue-to-fight-bill-which-hands-sacred-native-american-land-to-mining-company/208194/>


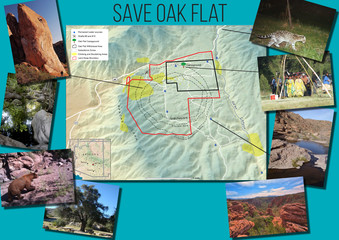


Source: <https://www.mintpressnews.com/arizona-apache-continue-to-fight-bill-which-hands-sacred-native-american-land-to-mining-company/208194/>


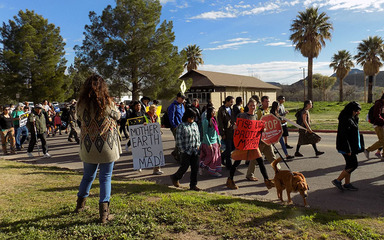


**Headline:** Resolution Copper (Rio Tinto and BHP) threatens to destroy Apache ceremonial grounds at Chi'chil Bildagoteel in Arizona for critical materials for the so-called energy transition

**Description:**

|  | Oak Flat is a sacred site in the homeland of the Apache Nation who know it as Chi’chil Biłdagoteel. It is also held sacred by Zuni, Yavapai, O’odham and Hopi people [14]. For centuries, people have visited Chi’chil Bildagoteel to hold ceremonies and to collect acorns and medicinal plants [8]. The area contains hundreds of petroglyphs, historic sites, and sacred springs [6][8][14]. The Apache have never lost their relationship with Chi’chil  Bildagoteel, although during some of their history the U.S. government has not allowed them to use the land [11][14].  The ceremonial grounds of Chi’chil Bildagoteel are threatened by the Resolution Copper Mine, a joint venture owned by Rio Tinto and BHP. The copper deposit is estimated to be the largest in North America, producing up to 25% of projected US demands [2].  Demand for copper is expected to rise as the energy industry transitions away from fossil fuels, and the auto industry begins manufacturing more electric vehicles [6].  Oak Flat is owned by the U.S. Forest Service, and was protected from mining in the 1950s, when it was listed as a Traditional Cultural Property on the National Register of Historic Places. The Resolution Copper mine was proposed in 2004, and in the following eight years more than a dozen congressional bills were proposed to transfer ownership of Oak Flat to Resolution Copper. Finally, in 2014 a rider transferring ownership of Oak Flat to the mining company was attached to the must-pass 2015 Defense Appropriations Act minutes before it was voted on in congress [6]. This last-minute provision granted 2,422 acres of the Tonto National Forest including the Oak Flat area to Resolution Copper in exchange for 5,344 acres of private land. A draft EIA for the project released in August 2019 detailed environmental, social and economic consequences so complex that it took the Forest Service more than 1,300 pages to lay them out [13]. The final EIA was released in January 2020, and the Trump administration approved the land swap. The non-profit Apache Stronghold filed a lawsuit contesting the land swap in 2019, and the incoming Biden administration withdrew the EIA in March 2020 for further evaluation [5].  In 2021 President Biden’s Department of Justice argued in favor of the land swap during court proceedings. Ruling on the case is expected in 2022. The Department of Justice argued in a legal brief that the protection of Oak Flat would require an act of Congress [4]. Arizona Representative Raul Grijalva has introduced the Save Oak Flat Act at least four times [9][10].  The Apache have established an encampment to protect Oak Flat with four crosses representing the entire surrounding sacred area, including its water, animals, oak trees, and other plants central to the tribal identity [11]. Apache Stronghold has led a multi-pronged effort with allies employing political, grassroots, artistic, nonprofit and media strategies [15] Regional Indigenous people have marched at least six times to protest the land swap. The Apache Stronghold camp has been targeted by vandals: on March 17, 2018, the four sacred crosses were ripped from the ground or destroyed [15].  The Resolution Copper deposit is more than 1,300 meters beneath the surface, and the company proposes to use block-caving to extract the ore: tunnels would be constructed beneath the surface, the ore is withdrawn, and the area above the deposit would collapse into a subsidence about 1.8 miles (3 km) long and 800-1115 feet (245-340 m) deep. The crushed ore would be hauled halfway to the surface — about 1,050 meters up (3,500 feet) — and shipped to a processing plant by conveyor. The EIA projects that Chi’chil Bildagoteel would be, “directly and permanently damaged” by the subsidence [1]. Tailings from the mine would occupy 2,000 to 6,000 acres (depending on the selected alternative for the tailings facility). The draft EIA states that between 14 and 16 sacred springs would be impacted by the loss of groundwater, [1] although other studies place the number at forty-six [14]. Burial sites are also likely to be impacted [1][14].  The EIA projects that the mine would consume more than 500,000 acre-feet (600,000,000 m3) of water from the Colorado River and from precious groundwater supplies, changing the flow of surrounding rivers and springs; mining waste could contaminate groundwater and streams [13]. The EIA also projects strains on municipal services in the nearby town of Superior and increases housing costs [13]. The mining would disturb the habitats of endangered species like the Western, yellow-billed cuckoo and the Southwestern willow flycatcher [13]. |
| --- | --- |

**Basic Data**

| Name of conflict: | Oak Flat copper and molybdenum mining, USA |
| --- | --- |
| Country: | [United States of America](https://ejatlas.org/country/united-states-of-america) |
| State or province: | Arizona |
| Location of conflict: | Superior, Pinal county |
| Accuracy of location | HIGH (Local level) |

**Source of Conflict**

| Type of conflict. 1st level: | Mineral Ores and Building Materials Extraction |
| --- | --- |
| Type of conflict. 2nd level: | Water access rights and entitlements Mineral ore exploration Tailings from mines Mineral processing |
| Specific commodities: | [Copper](https://ejatlas.org/commodity/copper) Molybdenum |

**Project Details and Actors**

| Project details | Total projection of 40 billion tons of copper produced over 40-50 years |
| --- | --- |
| Project area: | 4,050 to 7,080 hectares |
| Level of Investment for the conflictive project | $2,000,000,000 to date [2] |
| Type of population | Semi-urban |
| Affected Population: | Several Indigenous nations, other nearby residents |
| Start of the conflict: | 01/01/2004 |
| Company names or state enterprises: | [Resolution Copper Co.](https://ejatlas.org/company/pan-pacific-copper-co-ltd) from [United States of America](https://ejatlas.org/country-of-company/united-states-of-america) [Rio Tinto (Rio Tinto )](https://ejatlas.org/company/rio-tinto) from [United Kingdom](https://ejatlas.org/country-of-company/united-kingdom) [BHP Billiton (BHP)](https://ejatlas.org/company/bhp-billiton) from [United Kingdom](https://ejatlas.org/country-of-company/united-kingdom) |
| Relevant government actors: | USDA Forest Service Cooperating Agencies: Arizona Department of Environmental Quality, Arizona Department of Water Resources, Arizona Game and Fish Department, Arizona State Land Department, Arizona State Mine Inspector, Bureau of Land Management, Pinal County Air Quality Control District, U.S. Army Corps of Engineers, U.S. Environmental Protection Agency |
| Environmental justice organizations (and other supporters) and their websites, if available: | Apache Stronghold ([www.apache-stronghold.com](http://www.apache-stronghold.com/)), Endorsing organizations: National Congress of American Indians, National Center for American Indian Enterprise Development, Indian Land Tenure Association, Americans for Indian Opportunity, Coalition of Large Tribes, Midwest Alliance of Sovereign Tribes, United Southern and Eastern Tribes, Inter Tribal Association of Arizona, All Pueblo Council of Governors, Affiliated Tribes of Northwest Indians, Inter-Tribal Council of Nevada, Inc., Rocky Mountain Tribal Leaders Council, Great Plains Tribal Chairman’s Association, Native American Rights Fund, National Center for American Indian Enterprise Development, Indian Land Tenure Foundation, American Indians for Opportunity, Arizona Mining Reform Coalition, Concerned Citizens & Retired Miners Coalition, Center For Biological Diversity, National Wildlife Federation, Sierra Club, Access Fund, Earthworks, Patagonia, Inc., First Nations Heritage Protection Alliance, Outdoor Alliance, American Civil Liberties Union, Association on American Indian Affairs, HECHO, Baptist Joint Committee for Religious Liberty, Advisory Council on Historic Preservation, Center for American Progress, Teamsters Local Union 104 (Phoenix), Poor People's Campaign |

**Conflict & Mobilization**

| Intensity | MEDIUM (street protests, visible mobilization) |
| --- | --- |
| Reaction stage | PREVENTIVE resistance (precautionary phase) |
| Groups mobilizing: | Indigenous groups or traditional communities Local ejos Ethnically/racially discriminated groups Local scientists/professionals Religious groups San Carlos Apache, Zuni, Yavapai, O’odham and Hopi people |
| Forms of mobilization: | Artistic and creative actions (eg guerilla theatre, murals) Development of a network/collective action Lawsuits, court cases, judicial activism Media based activism/alternative media Objections to the EIA Official complaint letters and petitions Public campaigns Street protest/marches |

**Impacts**

| Environmental Impacts | **Potential:**Biodiversity loss (wildlife, agro-diversity), Loss of landscape/aesthetic degradation, Noise pollution, Soil contamination, Soil erosion, Waste overflow, Deforestation and loss of vegetation cover, Surface water pollution / Decreasing water (physico-chemical, biological) quality, Groundwater pollution or depletion, Reduced ecological / hydrological connectivity, Mine tailing spills, Desertification/Drought |
| --- | --- |
| Health Impacts | **Potential:**Accidents, Mental problems including stress, depression and suicide, Violence related health impacts (homicides, rape, etc..), Health problems related to alcoholism, prostitution |
| Socio-economical Impacts | **Potential:**Increase in violence and crime, Loss of livelihood, Loss of traditional knowledge/practices/cultures, Social problems (alcoholism, prostitution, etc..), Specific impacts on women, Loss of landscape/sense of place, Increase in Corruption/Co-optation of different actors, Land dispossession |

**Outcome**

| Project Status | Planned (decision to go ahead eg EIA undertaken, etc) |
| --- | --- |
| Conflict outcome / response: | Under negotiation Application of existing regulations Project temporarily suspended |
| Proposal and development of alternatives: | Not mining the area. The Save Oak Flat Act would revoke the land swap transferring ownership of Oak Flat to Resolution Copper |
| Do you consider this an environmental justice success? Was environmental justice served?: | No |
| Briefly explain: | Litigation is ongoing as of June 2022 |

**Sources & Materials**

| Juridical relevant texts related to the conflict (laws, legislations, EIAs, etc) | \| [1] USDA Forest Service, Resolution Copper Project and Land Exchange Draft Environmental Impact Statement, 2019 [[click to view]](https://www.resolutionmineeis.us/sites/default/files/deis/resolution-deis-executive-summary.pdf) \| \| --- \| \| [9] Save Oak Flat Act (US Congress public record) [[click to view]](https://www.congress.gov/bill/117th-congress/house-bill/1884) \| \| [12] National Defense Authorization Act for FY 2015, Section 3003, the Southeast Arizona Land Exchange and Conservation Act [[click to view]](https://www.congress.gov/bill/113th-congress/house-bill/3979) \| |
| --- | --- | --- | --- | --- |
| References to published books, academic articles, movies or published documentaries | \| [7] Lucas, Emily. The Environmental and Spiritual Significance of Chi’chil Bildagoteel, an Apache Sacred Site. Environmental program thesis, Colorado College, 5/2017 [[click to view]](http://users.neo.registeredsite.com/8/3/2/11897238/assets/Emily_Lucas_Official_T30056.pdf) \| \| --- \| \| [14] Welch, John R. (2017) Earth, Wind, and Fire: Pinal Apaches, Miners, and Genocide in Central Arizona, 1859-1874. Sage Open. [[click to view]](https://journals.sagepub.com/doi/full/10.1177/2158244017747016) \| |
|  | \| [2] Resolution Copper Project Overview. Company website \| \| --- \| \| [3] USDA Forest Service. Resolution Copper Update. [[click to view]](https://www.fs.usda.gov/detail/r3/home/?cid=FSEPRD858166) \| \| [4] Kelety, Josh. "Biden Administration Says Oak Flat Land Swap Should Proceed Despite Lawsuit", Phoenix New Times, 6/2/21 [[click to view]](https://www.phoenixnewtimes.com/news/biden-administration-says-oak-flat-land-swap-should-proceed-11558259) \| \| [5] Krol, Debra Utacia. "Biden official tours Oak Flat as Forest Service begins talks with tribes over copper mine", AZCentral, 2/13/22. [[click to view]](https://www.azcentral.com/story/news/local/arizona-environment/2022/02/11/biden-official-restarts-talks-tribes-over-oak-flat-copper-mine/9302271002/) \| \| [6] Oatman, Maddie. "EVs’ demand for copper escalates threat against Apache’s Oak Flat", High Country News. 4/20/22. [[click to view]](https://www.hcn.org/articles/mining-evs-demand-for-copper-escalates-threat-against-apaches-oak-flat) \| \| [8] Hedgpeth, Dana. "This land is sacred to the Apache, and they are fighting to save it", The Washington Post, 4/12/21. [[click to view]](https://www.washingtonpost.com/history/2021/04/12/oak-flat-apache-sacred-land/) \| \| [10] History of the Save Oak Flat Act (Apache Stronghold website) [[click to view]](http://apache-stronghold.com/save-oak-flat-act.html) \| \| [11] Apache Stronghold website [[click to view]](http://apache-stronghold.com/) \| \| [13] Whitman, Elizabeth. "New Report Provides Terrifying Detail of Mining Destruction Coming for Oak Flat." Phoenix New Times August 14, 2019 [[click to view]](https://www.phoenixnewtimes.com/news/resolution-copper-mine-rosemont-environmental-report-forest-service-11342414?fbclid=IwAR3NMTRV0QUdQPfCmanN9hS5xht7tzickb_LKwq99HEPhSAUYpHlYGw--Pg) \| \| [15] Sacred Land Film Project, November 2018 [[click to view]](https://sacredland.org/oak-flat-united-states/) \| \| Stern, Ray. "Environmental Study of Planned Copper Mine at Arizona's Oak Flat Could Halt Project", Phoenix New Times, March 22, 2016. [[click to view]](https://www.phoenixnewtimes.com/news/environmental-study-of-planned-copper-mine-at-arizonas-oak-flat-could-halt-project-8157637) \| \| Vice News 2017: Inside the Fight Against a Mining Development in Oak Flat [[click to view]](https://www.vice.com/en_ca/article/ypn5dw/inside-the-apache-stronghold-fighting-off-a-multinational-mining-company) \| |

**Meta information**

| Contributor: | ***Anonymous for this review*** |
| --- | --- |
| Last update | 23/06/2022 |
| Conflict ID: | 6041 |

**Log-Linear Regression**

Log-Linear Regression Loss of traditional knowledge/practices/cultures ✻ **Conflict Category**

| Model Coefficient | | | | | | | | | | | | | | | | | | |
| --- | --- | --- | --- | --- | --- | --- | --- | --- | --- | --- | --- | --- | --- | --- | --- | --- | --- | --- |
|  | | | | **95% Confidence Interval** | | | |  | | | | | | | | | | |
| **Predictor** | | **Estimate** | | **Lower** | | **Upper** | | **SE** | | **Z** | | **p** | | | **Rate ratio** | | | |
| Loss of traditional knowledge/practices/cultures ✻ **Biodiversity conservation conflicts:** |  |  |  |  |  |  |  |  |  |  |  | |  |  | |  |  |  |
| (0 – **1**) ✻ (0 – **1**) |  | 0.628 |  | 0.266 |  | 0.990 |  | 0.185 |  | 3.40 |  | | **< .001** |  | | 1.874 |  |  |
| Loss of traditional knowledge/practices/cultures ✻ **Biomass and Land Conflicts (Forests, Agriculture, Fisheries and Livestock Management):** |  |  |  |  |  |  |  |  |  |  |  | |  |  | |  |  |  |
| (0 – **1**) ✻ (0 – **1**) |  | 0.513 |  | 0.316 |  | 0.709 |  | 0.1003 |  | 5.11 |  | | **< .001** |  | | 1.670 |  |  |
| Loss of traditional knowledge/practices/cultures ✻ **Mineral Ores and Building Materials Extraction:** |  |  |  |  |  |  |  |  |  |  |  | |  |  | |  |  |  |
| (0 – **1**) ✻ (0 – **1**) |  | 0.439 |  | 0.268 |  | 0.610 |  | 0.0871 |  | 5.04 |  | | **< .001** |  | | 1.551 |  |  |
| Loss of traditional knowledge/practices/cultures ✻ **Waste Management:** |  |  |  |  |  |  |  |  |  |  |  | |  |  | |  |  |  |
| (0 – 1) ✻ (0 – 1) |  | -1.66 |  | -1.97 |  | -1.36 |  | 0.156 |  | -10.66 |  | | **< .001** |  | | 0.190 |  |  |
| Loss of traditional knowledge/practices/cultures ✻ **Water Management:** |  |  |  |  |  |  |  |  |  |  |  | |  |  | |  |  |  |
| (0 – 1) ✻ (0 – 1) |  | 0.510 |  | 0.307 |  | 0.713 |  | 0.1038 |  | 4.91 |  | | **< .001** |  | | 1.665 |  |  |

Log-Linear Regression Loss of traditional knowledge/practices/cultures ✻ **Commodities**

| Model Coefficients | | | | | | | | | | | | | | | | |
| --- | --- | --- | --- | --- | --- | --- | --- | --- | --- | --- | --- | --- | --- | --- | --- | --- |
|  | | | | **95% Confidence Interval** | | | |  | | | | | | | | |
| **Predictor** | | **Estimate** | | **Lower** | | **Upper** | | **SE** | | **Z** | | **p** | | **Rate ratio** | |  |
| Loss of traditional knowledge/practices/cultures ✻ **Carbon offsets**: |  |  |  |  |  |  |  |  |  |  |  |  |  |  |  |  |
| (0 – **1**) ✻ (0 – **1**) |  | 0.572 |  | 0.0667 |  | 1.077 |  | 0.258 |  | 2.22 |  | **0.026** |  | 1.771 |  |  |
| Loss of traditional knowledge/practices/cultures ✻ **Cellulose:** |  |  |  |  |  |  |  |  |  |  |  |  |  |  |  |  |
| (0 – **1**) ✻ (0 – **1**) |  | 0.735 |  | 0.163 |  | 1.306 |  | 0.292 |  | 2.52 |  | **0.012** |  | 2.085 |  |  |
| Loss of traditional knowledge/practices/cultures ✻ **Crude oil**: |  |  |  |  |  |  |  |  |  |  |  |  |  |  |  |  |
| (0 – **1**) ✻ (0 – **1**) |  | 0.459 |  | 0.224 |  | 0.695 |  | 0.1200 |  | 3.83 |  | **< .001** |  | 1.583 |  |  |
| Loss of traditional knowledge/practices/cultures ✻ **Gold:** |  |  |  |  |  |  |  |  |  |  |  |  |  |  |  |  |
| (0 – **1**) ✻ (0 – **1**) |  | 0.520 |  | 0.274 |  | 0.767 |  | 0.1257 |  | 4.14 |  | **< .001** |  | 1.683 |  |  |
| Loss of traditional knowledge/practices/cultures ✻ **Land:** |  |  |  |  |  |  |  |  |  |  |  |  |  |  |  |  |
| (0 – **1**) ✻ (0 – **1**) |  | 0.824 |  | 0.678 |  | 0.971 |  | 0.0747 |  | 11.04 |  | **< .001** |  | 2.281 |  |  |
| Loss of traditional knowledge/practices/cultures ✻ **Biological resources:** |  |  |  |  |  |  |  |  |  |  |  |  |  |  |  |  |
| (0 – **1**) ✻ (0 – **1**) |  | 0.572 |  | 0.215 |  | 0.929 |  | 0.182 |  | 3.14 |  | **0.002** |  | 1.772 |  |  |
| Loss of traditional knowledge/practices/cultures ✻ **Domestic municipal waste:** |  |  |  |  |  |  |  |  |  |  |  |  |  |  |  |  |
| (0 – **1**) ✻ (0 – **1**) |  | -1.52 |  | -1.841 |  | -1.20 |  | 0.163 |  | -9.31 |  | **< .001** |  | 0.219 |  |  |
| Loss of traditional knowledge/practices/cultures ✻ **Timber:** |  |  |  |  |  |  |  |  |  |  |  |  |  |  |  |  |
| (0 – **1**) ✻ (0 – **1**) |  | 0.988 |  | 0.619 |  | 1.358 |  | 0.1885 |  | 5.24 |  | **< .001** |  | 2.687 |  |  |
| Loss of traditional knowledge/practices/cultures ✻ **Ecosystem Services:** |  |  |  |  |  |  |  |  |  |  |  |  |  |  |  |  |
| (0 – **1**) ✻ (0 – **1**) |  | 0.900 |  | 0.454 |  | 1.345 |  | 0.227 |  | 3.96 |  | **< .001** |  | 2.459 |  |  |

 Log-Linear Regression Loss of traditional knowledge/practices/cultures ✻ **Affected groups**

| Model Coefficients | | | | | | | | | | | | | | | | |
| --- | --- | --- | --- | --- | --- | --- | --- | --- | --- | --- | --- | --- | --- | --- | --- | --- |
|  | | | | **95% Confidence Interval** | | | |  | | | | | | | | |
| **Predictor** | | **Estimate** | | **Lower** | | **Upper** | | **SE** | | **Z** | | **p** | | **Rate ratio** | |  |
| Loss of traditional knowledge/practices/cultures ✻ **Farmers:** |  |  |  |  |  |  |  |  |  |  |  |  |  |  |  |  |
| (0 – **1**) ✻ (0 – **1**) |  | 0.798 |  | 0.662 |  | 0.9349 |  | 0.0696 |  | 11.46 |  | **< .001** |  | 2.222 |  |  |
| Loss of traditional knowledge/practices/cultures ✻ **Fisher people:** |  |  |  |  |  |  |  |  |  |  |  |  |  |  |  |  |
| (0 – **1**) ✻ (0 – **1**) |  | 0.919 |  | 0.743 |  | 1.095 |  | 0.0897 |  | 10.2 |  | **< .001** |  | 2.506 |  |  |
| Loss of traditional knowledge/practices/cultures ✻ **Indigenous groups or traditional communities:** |  |  |  |  |  |  |  |  |  |  |  |  |  |  |  |  |
| (0 – **1**) ✻ (0 – **1**) |  | 2.008 |  | 1.850 |  | 2.166 |  | 0.0806 |  | 24.92 |  | **< .001** |  | 7.449 |  |  |
| Loss of traditional knowledge/practices/cultures ✻ **Landless peasants:** |  |  |  |  |  |  |  |  |  |  |  |  |  |  |  |  |
| (0 – **1**) ✻ (0 – **1**) |  | 0.838 |  | 0.606 |  | 1.069 |  | 0.1183 |  | 7.08 |  | **< .001** |  | 2.311 |  |  |
| Loss of traditional knowledge/practices/cultures ✻ **Pastoralists:** |  |  |  |  |  |  |  |  |  |  |  |  |  |  |  |  |
| (0 – **1**) ✻ (0 – **1**) |  | 0.965 |  | 0.660 |  | 1.269 |  | 0.1554 |  | 6.21 |  | **< .001** |  | 2.624 |  |  |
| Loss of traditional knowledge/practices/cultures ✻ **Ethnically/racially discriminated** groups: |  |  |  |  |  |  |  |  |  |  |  |  |  |  |  |  |
| (0 – **1**) ✻ (0 – **1**) |  | 0.849 |  | 0.666 |  | 1.031 |  | 0.0931 |  | 9.11 |  | **< .001** |  | 2.337 |  |  |
| Loss of traditional knowledge/practices/cultures ✻ **Religious groups:** |  |  |  |  |  |  |  |  |  |  |  |  |  |  |  |  |
| (0 – **1**) ✻ (0 – **1**) |  | 0.755 |  | 0.537 |  | 0.974 |  | 0.1115 |  | 6.78 |  | **< .001** |  | 2.128 |  |  |

  Log-Linear Regression Loss of traditional knowledge/practices/cultures ✻ **Mobilisation forms**

| Model Coefficients | | | | | | | | | | | | | | | |
| --- | --- | --- | --- | --- | --- | --- | --- | --- | --- | --- | --- | --- | --- | --- | --- |
|  | | | | **95% Confidence Interval** | | | |  | | | | | | | |
| **Predictor** | | **Estimate** | | **Lower** | | **Upper** | | **SE** | | **Z** | | **p** | | **Rate ratio** | |
| Loss of traditional knowledge/practices/cultures ✻ **Artistic and creative actions (e.g., guerilla theatre, murals):** |  |  |  |  |  |  |  |  |  |  |  |  |  |  |  |
| (0 – **1**) ✻ (0 – **1**) |  | 0.372 |  | 0.155 |  | 0.588 |  | 0.1105 |  | 3.37 |  | **< .001** |  | 1.450 |  |
| Loss of traditional knowledge/practices/cultures ✻ **Appeals/recourse to economic valuation of the environment:** |  | 0.892 |  | 0.682 |  | 1.102 |  | 0.1071 |  | 8.33 |  | **< .001** |  | 2.440 |  |
| (0 – **1**) ✻ (0 – **1**) |  |  |  |  |  |  |  |  |  |  |  |  |  |  |  |
| Loss of traditional knowledge/practices/cultures ✻ **Boycotts of official procedures:** |  |  |  |  |  |  |  |  |  |  |  |  |  |  |  |
| (0 – **1**) ✻ (0 – **1**) |  | 0.902 |  | 0.587 |  | 1.216 |  | 0.1604 |  | 5.62 |  | **< .001** |  | 2.464 |  |
| Loss of traditional knowledge/practices/cultures ✻ **Rights of nature arguments:** |  |  |  |  |  |  |  |  |  |  |  |  |  |  |  |
| (0 – **1**) ✻ (0 – **1**) |  | 1.20 |  | 0.991 |  | 1.417 |  | 0.1087 |  | 11.1 |  | **< .001** |  | 3.333 |  |
| Loss of traditional knowledge/practices/cultures ✻ **Refusal of compensation:** |  |  |  |  |  |  |  |  |  |  |  |  |  |  |  |
| (0 – **1**) ✻ (0 – **1**) |  | 1.29 |  | 0.949 |  | 1.63 |  | 0.1745 |  | 7.40 |  | **< .001** |  | 3.637 |  |
| Loss of traditional knowledge/practices/cultures ✻ **Land occupation:** |  |  |  |  |  |  |  |  |  |  |  |  |  |  |  |
| (0 – **1**) ✻ (0 – **1**) |  | 0.669 |  | 0.452 |  | 0.886 |  | 0.1108 |  | 6.04 |  | **< .001** |  | 1.952 |  |

Log-Linear Regression Loss of traditional knowledge/practices/cultures ✻ **Outcomes**

|  | | | | **95% Confidence Interval** | | | |  |  |  |  |  |  |  |
| --- | --- | --- | --- | --- | --- | --- | --- | --- | --- | --- | --- | --- | --- | --- |
| **Predictor** | | **Estimate** | | **Lower** | | **Upper** | | **SE** | | **Z** | | **p** | | **Rate ratio** |
| Loss of traditional knowledge/practices/cultures ✻ **Criminalization of activists:** |  |  |  |  |  |  |  |  |  |  |  |  |  |  |
| (0 – **1**) ✻ (0 – **1**) |  | 0.608 |  | 0.439 |  | 0.777 |  | 0.0863 |  | 7.05 |  | **< .001** |  | 1.837 |
| Loss of traditional knowledge/practices/cultures ✻ **Assassinations:** |  |  |  |  |  |  |  |  |  |  |  |  |  |  |
| (0 – **1**) ✻ (0 – **1**) |  | 0.723 |  | 0.507 |  | 0.938 |  | 0.1097 |  | 6.58 |  | **< .001** |  | 2.060 |
| Loss of traditional knowledge/practices/cultures ✻ **Land demarcation:** |  |  |  |  |  |  |  |  |  |  |  |  |  |  |
| (0 – **1**) ✻ (0 – **1**) |  | 1.01 |  | 0.702 |  | 1.323 |  | 0.1584 |  | 6.39 |  | **< .001** |  | 2.752 |
| Loss of traditional knowledge/practices/cultures ✻ **Migration/displacement:** |  |  |  |  |  |  |  |  |  |  |  |  |  |  |
| (0 – **1**) ✻ (0 – **1**) |  | 0.836 |  | 0.665 |  | 1.008 |  | 0.0875 |  | 9.55 |  | **< .001** |  | 2.308 |
| Loss of traditional knowledge/practices/cultures ✻ **Repression:** |  |  |  |  |  |  |  |  |  |  |  |  |  |  |
| (0 – **1**) ✻ (0 – **1**) |  | 0.682 |  | 0.518 |  | 0.846 |  | 0.0837 |  | 8.15 |  | **< .001** |  | 1.978 |
| Loss of traditional knowledge/practices/cultures ✻ **Fostering a culture of peace:** |  |  |  |  |  |  |  |  |  |  |  |  |  |  |
| (0 – **1**) ✻ (0 – **1**) |  | 0.878 |  | 0.361 |  | 1.395 |  | 0.264 |  | 3.33 |  | **< .001** |  | 2.406 |

**Margin of error**

**SE/ √ (sample size) * z-score**

| **Conflict Category** | **z-score** | **SE** | **Sample size** | **SE of the mean** | **Margin of error** | **Margin of error %** |
| --- | --- | --- | --- | --- | --- | --- |
| Traditional knowledge/practices/cultures ✻ **Biodiversity conservation conflicts** | 3,4 | 0,185 | 139 | 0,015691495 | 0,053351084 | **5,34** |
| Traditional knowledge/practices/cultures ✻ **Biomass and Land Conflicts** (Forests, Agriculture, Fisheries and Livestock Management): | 5,11 | 0,1003 | 497 | 0,00449907 | 0,022990247 | **2,30** |
| Traditional knowledge/practices/cultures ✻ **Mineral Ores and Building Materials Extraction** | 5,04 | 0,0871 | 686 | 0,003325493 | 0,016760487 | **1,68** |
| Traditional knowledge/practices/cultures ✻ **Waste Management** | 10,66 | 0,156 | 276 | 0,009390097 | 0,100098429 | **10,01** |
| Traditional knowledge/practices/cultures ✻ **Water Management** | 4,91 | 0,1038 | 459 | 0,004844969 | 0,023788798 | **2,38** |
|  |  |  |  |  |  |  |
| **Commodities** | **z-score** | **SE** | **Sample size** | **SE of the mean** | **Margin of error** | **Margin of error %** |
| Traditional knowledge/practices/cultures ✻ **Carbon offsets** | 2,22 | 0,258 | 69 | 0,03105955 | 0,068952201 | **6,90** |
| Traditional knowledge/practices/cultures ✻ **Cellulose** | 2,52 | 0,292 | 57 | 0,038676345 | 0,097464389 | **9,75** |
| Traditional knowledge/practices/cultures ✻ **Crude oil** | 3,83 | 0,12 | 328 | 0,006625892 | 0,025377165 | **2,54** |
| Traditional knowledge/practices/cultures ✻ **Gold** | 4,14 | 0,1257 | 301 | 0,007245228 | 0,029995242 | **3,00** |
| Traditional knowledge/practices/cultures ✻ **Land** | 11,04 | 0,0747 | 1159 | 0,002194214 | 0,024224117 | **2,42** |
| Traditional knowledge/practices/cultures ✻ **Biological resources** | 3,14 | 0,182 | 140 | 0,015381807 | 0,048298875 | **4,83** |
| Traditional knowledge/practices/cultures ✻ **Domestic municipal waste** | 9,31 | 0,163 | 233 | 0,010678485 | 0,099416696 | **9,94** |
| Traditional knowledge/practices/cultures ✻ **Timber** | 5,24 | 0,1885 | 154 | 0,015189763 | 0,079594357 | **7,96** |
| Traditional knowledge/practices/cultures ✻ **Ecosystem Services** | 3,96 | 0,227 | 101 | 0,022587344 | 0,089445883 | **8,94** |
|  |  |  |  |  |  |  |
| **Affected groups** | **z-score** | **SE** | **Sample size** | **SE of the mean** | **Margin of error** | **Margin of error %** |
| Traditional knowledge/practices/cultures ✻ **Farmers:** | 11,46 | 0,0696 | 1550 | 0,001767842 | 0,020259467 | **2,03** |
| Traditional knowledge/practices/cultures ✻ **Fisher people** | 10,2 | 0,0897 | 726 | 0,003329079 | 0,033956608 | **3,40** |
| Traditional knowledge/practices/cultures ✻ **Indigenous groups or traditional communities** | 24,92 | 0,0806 | 1425 | 0,002135146 | 0,053207842 | **5,32** |
| Traditional knowledge/practices/cultures ✻ **Landless peasants** | 7,08 | 0,1183 | 380 | 0,006068662 | 0,042966127 | **4,30** |
| Traditional knowledge/practices/cultures ✻ **Pastoralists** | 6,21 | 0,1554 | 226 | 0,010337054 | 0,064193107 | **6,42** |
| Traditional knowledge/practices/cultures ✻ **Ethnically/racially discriminated groups** | 9,11 | 0,0931 | 650 | 0,003651682 | 0,033266827 | **3,33** |
| Traditional knowledge/practices/cultures ✻ **Religious groups** | 6,78 | 0,1115 | 420 | 0,005440643 | 0,036887559 | **3,69** |
|  |  |  |  |  |  |  |
| **Forms of mobilization** | **z-score** | **SE** | **Sample size** | **SE of the mean** | **Margin of error** | **Margin of error %** |
| Traditional knowledge/practices/cultures ✻ **Artistic and creative actions** | 3,37 | 0,1105 | 386 | 0,005624302 | 0,018953897 | **1,90** |
| Traditional knowledge/practices/cultures ✻ **Appeals to economic valuation of the environment** | 8,33 | 0,1071 | 481 | 0,00488334 | 0,040678219 | **4,07** |
| Traditional knowledge/practices/cultures ✻ **Boycotts of official procedures** | 5,62 | 0,1604 | 206 | 0,011175598 | 0,062806858 | **6,28** |
| Traditional knowledge/practices/cultures ✻ **Arguments for the rights of mother nature** | 11,1 | 0,1087 | 529 | 0,004726087 | 0,052459565 | **5,25** |
| Traditional knowledge/practices/cultures ✻ **Refusal of compensation** | 7,4 | 0,1745 | 210 | 0,012041644 | 0,089108166 | **8,91** |
| Traditional knowledge/practices/cultures ✻ **Land occupation** | 6,04 | 0,1108 | 414 | 0,005445523 | 0,032890958 | **3,29** |
|  |  |  |  |  |  |  |
| **Outcomes** | **z-score** | **SE** | **Sample size** | **SE of the mean** | **Margin of error** | **Margin of error %** |
| Traditional knowledge/practices/cultures ✻ **Criminalization of activists** | 7,05 | 0,0863 | 731 | 0,003191921 | 0,022503042 | **2,25** |
| Traditional knowledge/practices/cultures ✻ **Assassinations** | 6,58 | 0,1097 | 430 | 0,005290204 | 0,034809541 | **3,48** |
| Traditional knowledge/practices/cultures ✻ **Land demarcation** | 6,39 | 0,1584 | 222 | 0,010631112 | 0,067932805 | **6,79** |
| Traditional knowledge/practices/cultures ✻ **Migration/displacement** | 9,55 | 0,0875 | 751 | 0,00319292 | 0,030492389 | **3,05** |
| Traditional knowledge/practices/cultures ✻ **Repression** | 8,15 | 0,0837 | 806 | 0,002948207 | 0,024027885 | **2,40** |
| Traditional knowledge/practices/cultures ✻ **Fostering a culture of peace** | 3,33 | 0,264 | 74 | 0,030689377 | 0,102195624 | **10,22** |
